# Comprehensive Lipidome Profiling of the Kidney in Early-Stage Diabetic Nephropathy

**DOI:** 10.3389/fendo.2020.00359

**Published:** 2020-06-19

**Authors:** Biyu Hou, Ping He, Peng Ma, Xinyu Yang, Chunyang Xu, Sin Man Lam, Guanghou Shui, Xiuying Yang, Li Zhang, Guifen Qiang, Guanhua Du

**Affiliations:** ^1^State Key Laboratory of Bioactive Substance and Function of Natural Medicines, Institute of Materia Medica, Peking Union Medical College, Beijing Key Laboratory of Drug Target, Screening Research, Chinese Academy of Medical Sciences, Beijing, China; ^2^Beijing Obstetrics and Gynecology Hospital, Capital Medical University, Beijing, China; ^3^Institute of Genetics and Developmental Biology, Chinese Academy of Sciences, Beijing, China

**Keywords:** diabetic nephropathy, lipidomics, glomerular filtration barrier, lipotoxicity, sphingolipids

## Abstract

Metabolic changes associated with diabetes are reported to lead to the onset of early-stage diabetic nephropathy (DN). Furthermore, lipotoxicity is implicated in renal dysfunction. Most studies of DN have focused on a single or limited number of lipids, and the lipidome of the kidney during early-stage DN remains to be elucidated. In the present study, we aimed to comprehensively identify lipid abnormalities during early-stage DN; to this end, we established an early-stage DN rat model by feeding a high-sucrose and high-fat diet combined with administration of low-dose streptozotocin. Using a high-coverage, targeted lipidomic approach, we established the lipid profile, comprising 437 lipid species and 25 lipid classes, of the kidney cortex in normal rats and the DN rat model. Our findings additionally confirmed that the DN rat model had been successfully established. We observed distinct lipidomic signatures in the DN kidney, with characteristic alterations in side chain composition and degree of unsaturation. Glyceride lipids, especially cholesteryl esters, showed a significant increase in the DN kidney cortex. The levels of most phospholipids exhibited a decline, except those of phospholipids with side chain of 36:1. Furthermore, the levels of lyso-phospholipids and sphingolipids, including ceramide and its derivatives, were dramatically elevated in the present DN rat model. Our findings, which provide a comprehensive lipidome of the kidney cortex in rats with DN, are expected to be useful for the identification of pathologically relevant lipid species in DN. Furthermore, the results represent novel insights into the mechanistic basis of DN.

## Introduction

Diabetes mellitus (DM) is a metabolic disorder characterized by hyperglycemia and is accompanied by an increased risk of macrovascular and microvascular complications (1). As one of the most common microvascular complications of diabetes, diabetic nephropathy (DN) is characterized by altered glomerular filtration and proteinuria, resulting in end-stage renal disease (ESRD), which makes timely diagnosis and prevention critical (2). Although hyperglycemia and hypertension are known to drive the onset and progression of DN, intensive glycemic control has only modest effects and fails to stop DN progression to ESRD and death (3, 4). Therefore, elucidation of the mechanisms underlying DN and the development of effective therapeutic strategies are essential for the prevention of renal dysfunction (5).

Abnormal lipid metabolism is associated with prediabetes, type 1, and type 2 DM (T2DM). Metabolic changes induced by diabetes lead to glomerular hypertrophy, glomerulosclerosis, tubulointerstitial inflammation, and fibrosis. Renal lipotoxicity has been implicated in renal dysfunction as well as several pathological hallmarks of DN patients (4, 6, 7). As important biomolecules, lipids have many essential biological functions, which can be identified and quantified by lipidomics (1). In studies over the last few decades, lipidomic analyses have been performed to investigate biomarkers indicative of DN progression. Zhu et al. performed normal-phase liquid chromatography coupled with time-of-flight mass spectrometry (NPLC-TOF/MS) to analyze plasma phospholipids in T2DM and DN patients, and we identified two new biomarkers that distinguish healthy individuals, T2DM, and DN patients (8). Zhao et al. used gas chromatography (GC)/TOF/MS in tandem to analyze the effect of the Chaihuang–Yishen formula on the lipidome in progressive DN induced by uninephrectomy combined with streptozotocin (STZ) injection (9). Most previous studies have focused on a single or a limited number of lipids and lack analysis of the side chains. Sas et al. found the negative correlation with levels of ceramides (Cer) C16:0 and C24:1 in plasma and kidney tissue in diabetic mice (6). Zhao et al. focused on several kinds of phospholipids and sphingomyelins in progressive DN (9), whereas Kumari performed an integrated lipidomic analysis of urinary exosomes in DN patients to identify glycerol lipids that may be involved in phospholipid and sphingolipid metabolism (10). Chen et al. reported that perturbations in fatty acid and triglyceride (TG) metabolism are strongly correlated with phospholipids in patients with advanced chronic kidney disease (11). However, a comprehensive lipidomic analysis of early-stage DN has not been reported to date, and the physiological and molecular mechanisms associated with DN development remain to be identified.

In this study, we established an early-stage DN rat model by feeding rats a high-sucrose and high-fat diet (HFD) combined with STZ injection. We found that diabetic rats exhibited early-stage DN symptoms with microalbuminuria, injured renal function, basement membrane thickening, and glomerular hypertrophy. Further, using a high-coverage and targeted lipidomic approach, we identified a comprehensive lipidome of the kidney cortex, comprising 437 lipid species and 25 lipid classes. We additionally sought to identify pathologically relevant lipid species via comparison of the kidney lipidome between normal and diabetic rats. The findings offer new insights into the mechanistic basis of DN and may be useful for the development of potential novel therapeutic strategies against this disease.

## Materials and Methods

### Reagents and Internal Standards

STZ was purchased from Sigma Chemical Co, USA. Chloroform and methanol were purchased from Merck (Merck Pte. Ltd., China). d5-Triacylglycerol (TAG)(16:0)3, d5-TAG(14:0)3, d5-TAG(18:0)3, d5-diacylglycerol (DAG)(1,3-16:0), d5-DAG(1,3-18:1), and cholesteryl-2,2,3,4,4,6-d6 octadecanoate cholesterol-26,26,26,27,27,27-d6 were obtained from Avanti Polar Lipids (Alabaster, AL, USA). Phosphatidylinositol (PI)-d31(16:0/18:1) was obtained from Echelon Biosciences, Inc. (Salt Lake City, UT). Phosphatidylcholine (PC)-d31(16:0/18:1), phosphatidylethanolamine (PE)-d31(16:0/18:1), phosphatidylserine (PS)-d31(16:0/18:1), phosphatidic acid (PA)-d31(16:0/18:1), PA(17:0/17:0), phosphatidylglycerol (PG)-d31(16:0/18:1), lyso-bisphosphatidic acid (LBPA)-(14:0/14:0), lyso-PC(LPC)-17:0, lyso-PE(LPE)-17:1, lyso-PS(LPS)-17:1, Cer-d18:1/17:0, glucosylceramide (GluCer)-d18:1/8:0, and galactosylceramide (GalCer)-d18:1/8:0, d31-16:0, and d8-20:4 were obtained from Avanti Polar Lipids (Alabaster, AL).

### Animal Experiments

#### Animals

All animal experiments were approved by the Animal Care Committee of the Institute of Materia Medica, Chinese Academy of Medical Sciences. Male Sprague–Dawley (SD) rats (130–150 g) were provided by Beijing HFK Bioscience Co. Ltd. (Beijing, China) and housed in a temperature-controlled and humidity-controlled specific-pathogen free (SPF) barrier system, with a 12-h light/12-h dark cycle.

#### Establishment of Diabetic Nephropathy Rat Model

The DN rat model was induced as previously described (2). Briefly, after feeding with a high-sucrose and HFD for 4 weeks (standard diet supplemented with 10% sucrose, 10% lard stearin, 2% cholesterin, and 0.5% cholic acid), rats were intraperitoneally injected with 30 mg/kg of STZ dissolved in 0.1 M of citrate buffer (pH 4.4). Three days after injection, rats with fasting blood glucose (FBG) levels between 10 and 20 mM were identified as diabetic rats and selected for continued feeding of HFD (10% lard stearin, 2% cholesterin, and 0.5% cholic acid) for another 8 weeks (DN group). An age-matched control group (NC group) was injected with citrate buffer and fed a normal diet throughout the duration of the experiment. Each group contained six rats. FBG levels were measured using an ACCU-CHEK® active glucometer (Roche). Serum fructosamine, blood TGs, and total cholesterol (TCHO) were detected with an automatic analyzer (TOSHIBA Acute TBA-40FR, TOSHIBA, Tokyo, Japan) at the end of the experimental period.

#### Renal Function Analysis

At the eighth week, we performed 24-h urine collection from rats housed in metabolism cages with free access to water and food. The kidney coefficient was calculated as the ratio of kidney weight to body weight. Twenty-four-hour urinary albumin was assessed by ELISA assay (CUSABIO, Wuhan, China). Urinary creatinine (CR) levels were determined using a commercial assay kit (Jiancheng Biotech Co., Ltd., Nanjing, China). CR clearance (Ccr) was calculated using the formula: Ccr (μl/min) = (Ucr/Pcr) × urine volume (μl/min), as previously described (2, 12). After completion of the experiments, rats were humanely sacrificed, and blood samples were collected. Serum CR, blood urea nitrogen (BUN), and serum *N*-acyl-β-glucosidase (NAG) activity was assessed in accordance with the manufacturer's instructions.

#### Renal Histopathology Analysis

Rats were humanely sacrificed, and their left kidneys were fixed in 10% (w/v) neutral formaldehyde, dehydrated with a graded series of alcohol, and embedded in paraffin wax. Paraffin sections (4 μm thick) of kidney were subjected to hematoxylin–eosin (HE), periodic acid-Schiff (PAS), and periodic acid-silver methenamine (PASM) staining following the standard staining protocols. The sections were imaged with a microscope (Nikon Eclipse Ti-U, Nikon Corporation, Tokyo, Japan).

Dewaxed sections were blocked with 5% bovine serum albumin (BSA) after antigen retrieval. Sections were then incubated with primary antibody against CD68 (1:100; CST, USA) followed by incubation with horseradish peroxidase (HRP)-conjugated goat anti-rabbit IgG (Dako, Wuhan, China). CD68 expression was visualized by diaminobenzidine (DAB) (Dako, Wuhan, China) staining. The sections were then imaged with a microscope (Nikon Eclipse Ti-U, Nikon Corporation, Tokyo, Japan).

### Kidney Cortex Lipidome Analysis

#### Sample Preparation and Lipid Extraction

Prior to tissue collection, kidneys were perfused to exclude the possible interference from the blood. Frozen kidney cortex tissue was deactivated with a 900-μl mixture of chloroform:methanol (1:2) containing 10% deionized H_2_O. Finely cut pieces were homogenized and incubated for 1 h at 4°C. Next, 300 μl of chloroform and 400 μl of deionized H_2_O were added to the sample. The mixture was vortexed vigorously for 1 min and centrifuged for 5 min at 12,000 rpm, at 4°C. The organic phase in the lower layer was transferred to a new tube, and 500 μl of chloroform was added for another extraction step. The combined extracts were dried using a SpeedVac (Geneva, UK) and stored at −80°C for further analysis.

#### Quantitative Lipidomic Analysis

A comprehensive lipidomic platform based on an Exion ultra-performance liquid chromatography (UPLC) coupled with Sciex QTRAP 6500 Plus was used. UPLC-MS/MS analyses were conducted in electrospray ionization (ESI) mode, with conditions as follows: curtain gas = 20, ion spray voltage = 5,500 V, temperature = 400°C, ion source gas 1 = 35, and ion source gas 2 = 35. Briefly, polar lipids were separated using a Phenomenex Luna 3 μm of silica column (inner diameter 150 × 2.0 mm) with two mobile phases: mobile phase A (chloroform:methanol:ammonium hydroxide, 89.5:10:0.5) and mobile phase B (chloroform:methanol:ammonium hydroxide:water, 55:39:0.5:5.5). Gradient separation was conducted as follows: the gradient was maintained with 95% A for 5 min and then linearly reduced to 60% in 7 min and held for 4 min, after which it declined to 30% and was then held for 15 min; finally, the original gradient was applied and maintained for 5 min. PC-d31(16:0/18:1), PEd31(16:0/18:1), PS-d31(16:0/18:1), PI-d31(16:0/18:1), PA-d31(16:0/18:1), PA(17:0/17:0), PG-d31(16:0/18:1), LBPA-(14:0/14:0), LPC-17:0, LPE-17:1, LPS-17:1, Cer-d18:1/17:0, GluCer-d18:1/8:0, and GalCer-d18:1/8:0, d31-16:0, and d8-20:4 were used as internal standards to spike individual lipids. Separation of TAGs and DAGs was carried out with modified reversed-phase HPLC/ESI/MS/MS, as described previously, with a Phenomenex Kinetex 2.6 μm of C18 column (4.6 × 100 mm) (13). Isocratic elution was used to separate lipids with a mobile phase of chloroform:methanol:0.1 M ammonium acetate (100:100:4), at a flow rate of 160 μl/min for 20 min. d5-TAG(16:0)3, d5-TAG(14:0)3, and d5-TAG(18:0)3 were used to spike individual TAG. d5-DAG(1,3-16:0) and d5-DAG(1,3-18:1) were used to spike individual DAG based on Neutral miss MS/MS technology. Free cholesterol (Cho), free fatty acids (FFAs), sterols, and their esters were analyzed by HPLC–MS–atmospheric pressure chemical ionization (APCI) mode with a Phenomenex Kinetex column of 2.6 μm of C18 (4.6 × 100 mm).

### Statistical Analysis

Data are presented as means ± standard error of the mean (SEM). Differences between NC and DN groups were determined by statistical analysis using the unpaired two-tailed Student's *t*-test. Principal component analysis (PCA) was implemented using MetaboAnalyst (http://www.metaboanalyst.ca/). Data were auto-scaled before analysis. A value of *P* < 0.05 was considered to indicate statistically significant results.

## Results

### Confirmation of the Rat Model of Early-Stage Diabetic Nephropathy

**Figure 1:**
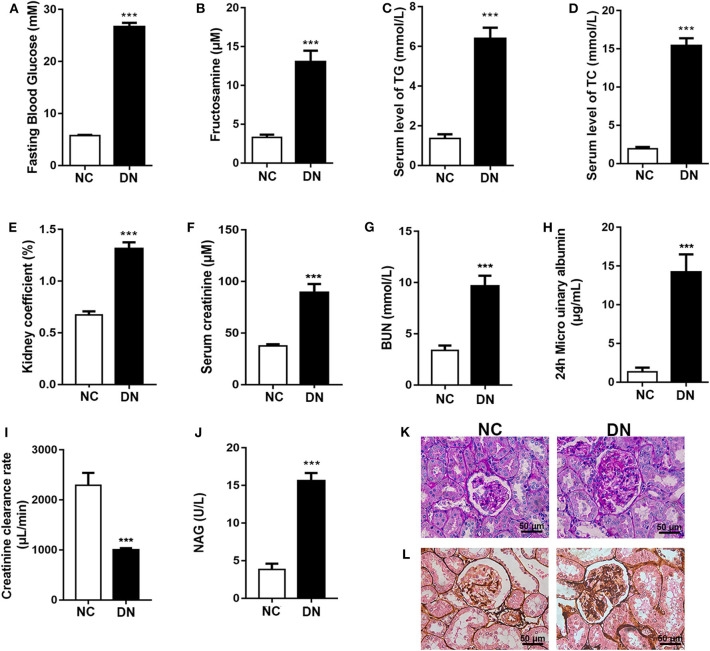
Confirmation of the early-stage diabetic nephropathy (DN) rat model. After diabetic nephropathy was induced by feeding high-fat diet (HFD) combined with injection of low-dose streptozotocin (STZ) for 8 weeks, **(A)** fasting blood glucose; **(B)** fructosamine; serum level of **(C)** TG and **(D)** TCHO; **(E)** kidney coefficient; serum level of **(F)** creatinine and **(G)** BUN; **(H)** 24-h microurinary albumin; **(I)** creatinine clearance; and **(J)** *N*-acyl-β-glucosidase (NAG) activity were determined. **(K)** Periodic acid-Schiff (PAS) staining and **(L)** periodic acid-silver methenamine (PASM) staining were conducted to examine renal structural changes. Data are presented as means ± SEM, *n* = 6 per group; ***P < 0.001.

Eight weeks of HFD feeding combined with low-dose STZ injection resulted in the induction of elevated blood glucose and fructosamine levels in the DN group of rats (Figures 1A,B). Furthermore, serum lipid levels were significantly increased, as evidenced by elevated TG and TCHO (Figures 1C,D). As shown in Figures 1E–G, the kidneys in the DN group exhibited hypertrophy (ratio of kidney weight to body weight). Significantly increased CR and BUN indicated damaged glomerular filtration function. The 24-h urinary albumin levels in DN rats increased to 14.21 μg/ml, and the Ccr rate declined to 999.26 μl/min (Figures 1H,I). In addition, elevated NAG suggested compromised tubular function in diabetic rats (Figure 1J). Further, we observed increased glomerular size in DN rats and mesangial expansion with increased red staining areas in diabetic glomeruli stained with PAS (Figure 1K). PASM staining revealed that basement membranes in DN rats were thickened in comparison with those of the NC group (Figure 1L); this phenomenon is indicative of early-stage DN. These findings confirm the successful induction of early-stage DN in the rat model.

### General Lipid Composition of the Kidney Cortex in Early-Stage Diabetic Nephropathy

**Figure 2:**
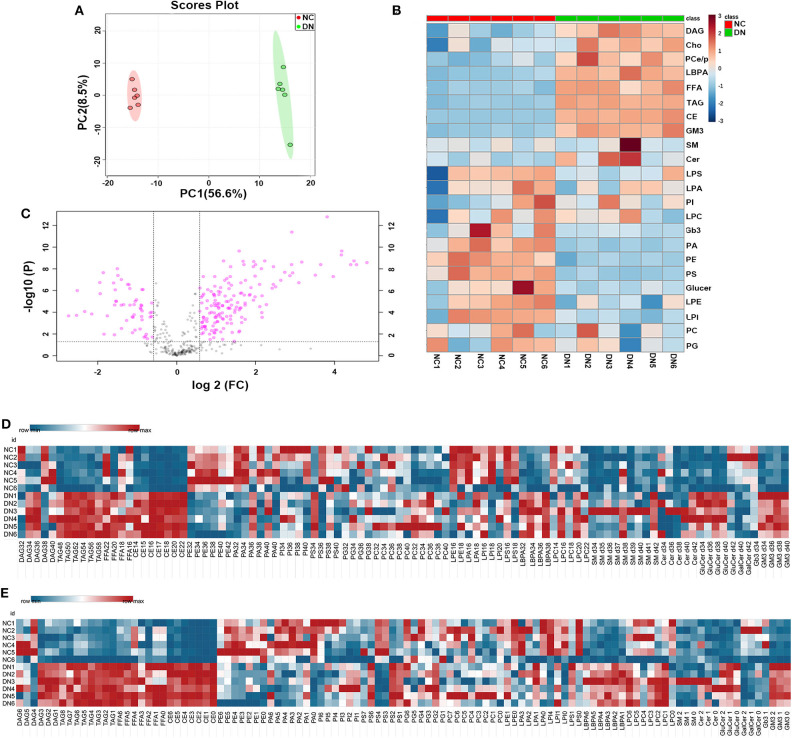
General analysis of lipidomic profiles in the kidney cortex of diabetic nephropathy (DN) and normal rats. **(A)** Principal component analysis (PCA) of whole lipidome of the two groups. **(B)** Volcano plot of all lipid species detected in the two groups. Data are shown as ratios of DN/NC, false discovery rate (FDR) < 0.05, and fold change > 1.5 is highlighted. Heatmap analysis of **(C)** main lipid class, **(D)** overall enrichment of chain length for lipid species, and **(E)** overall quantification of unsaturated bonds for lipid species; *n* = 6 per group.

High-coverage, targeted lipidomic analysis of the kidney cortex revealed 437 lipid species and 25 classes (see Supplementary Material). PCA of the whole lipidome showed clear differences between the data for the NC group and the DN group, suggesting a contrasting lipidome signature between normal and DN (Figure 2A). Detailed changes in lipid class are shown in Figure 2B, with a distinguishing pattern observed for glycerides and phospholipids as well as sphingolipids. Glycerides, including TAG, DAG, FFAs, Cho, and cholesteryl esters (CE), were significantly increased in the kidneys of the DN group. Interestingly, the levels of some phospholipids, such as PSs, were decreased; and those of lyso-phospholipids, such as LBPAs, were increased in the DN group. Volcano plot analysis with false discovery rate (FDR) < 0.05 and fold change (FC) > 1.5 was performed using Student's *t*-test after quality control. Of the 149 species that were differentially expressed, 114 species from 11 classes were significantly elevated; most of these belonged to TAG, CE, and LBPA classes. Among the lipid species that exhibited a significant decrease in levels, phospholipids, including PE, were predominant (Figure 2C). Heatmap analysis of overall lipid species in terms of chain length and unsaturated bonds showed a similar alteration pattern with the total quantities of lipid classes. However, some specific lipid species, such as DAG with 38C or DAG with four unsaturated bonds, and PS 34C or PS with one to two unsaturated bonds, showed different alterations as compared with TAG, CE, and LBPA (Figures 2D,E).

### Homogeneous Increases in Glyceride and Sterol Lipids in Early-Stage Diabetic Nephropathy

**Figure 3:**
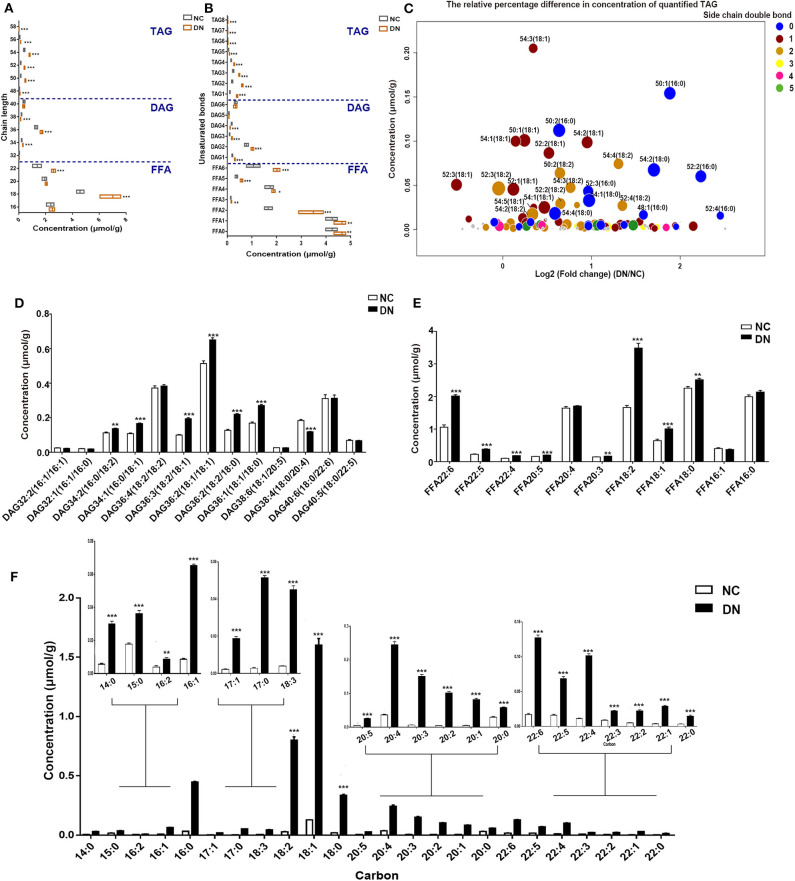
Differences in glycerides, fatty acids, and sterol lipids between normal and diabetic nephropathy (DN) rats. **(A)** Changes in the numbers of total carbons were analyzed for free fatty acid (FFA), diacylglycerol (DAG), and triacylglycerol (TAG) total concentrations. **(B)** Analysis of the changes in the number of unsaturated bonds in FFA, DAG, and TAG lipid species. **(C)** Bubble map showing the differences in TAG species content between the two groups, with the sizes of circles representing the level of significance calculated by –log 2 (*P*), colored by the number of unsaturated side chains. The content of DAG species **(D)**, FFA species **(E)**, and CE species **(F)** was analyzed. Data are presented as means ± SEM, *n* = 6 per group; *P < 0.05, **P < 0.01, ***P < 0.001.

Analysis of the changes in total carbon and unsaturated bonds of glycerides showed that the most drastic alterations, representing a 2.9- to 3.3-fold increase, were observed in TAG with 50–52C in DN rats. However, the content was lower than that of DAG and FFA (Figure 3A). Interestingly, DAG with 38C was the only glyceride species in DN kidneys to exhibit a decrease, with a 30% reduction observed in comparison with that in normal rats. TAG and FFA with two unsaturated bonds showed the most marked increase, whereas DAG with four unsaturated bonds showed a significant decrease (Figure 3B). Because TAG was the most altered glyceride lipid class, we analyzed the concentration and relative differences in its side chains (Figure 3C). The horizontal axis of the bubble plot indicates the FC of TAG (DN/NC), whereas the vertical axis indicates the concentration of TAG lipid species in the DN kidney cortex. Each dot represents a TAG species and was assigned a color on the basis of the number of unsaturation bonds in its side chain. The size of each dot represents the –log_2_ (*P*-value) obtained by comparing the NC and DN groups. As shown in Figure 3C, the majority of dots were distributed to the right side of the plot with log_2_ (FC) larger than 0, indicating that most TAG species were increased in the kidney cortex of DN. TAG with side chains containing one to two unsaturated bonds (blue dots and orange dots) were the major components of the overall TAG class. TAG with unsaturated side chains including 18:0 and 16:0, especially TAG 50:1(16:0), TAG 52:2(16:0), and TAG 52:4(16:0), showed the most marked alterations. Both DAG and FFA with 18C were significantly increased in DN kidneys (Figures 3D,E). In the CE class, each CE species was significantly elevated, particularly CE with 18C side chains, including 18:2, 18:1, and 18:0, with a 12- to 28-fold increase relative to normal rats (Figure 3F). Collectively, neutral lipid content; increased unsaturation for TAG, DAG, and fatty acids; and a homogeneous increase in CE were observed in the cortex of early-stage DN kidneys.

### Remodeling of Phospholipids in Early-Stage Diabetic Nephropathy

**Figure 4:**
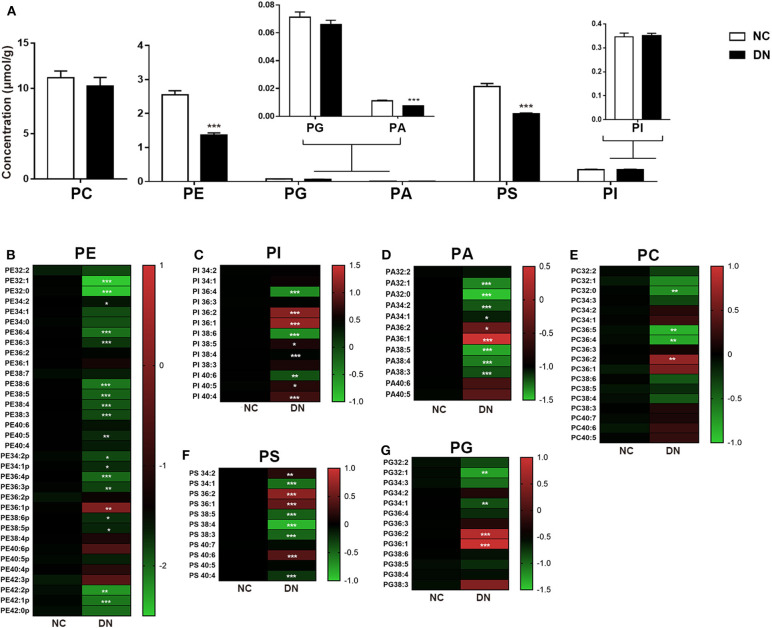
Phospholipid remodeling in the kidney cortex of diabetic nephropathy (DN) and normal rats. **(A)** Total content of each phospholipid class. Ratio heat plot analysis of **(B)** phosphatidylethanolamine (PE), **(C)** phosphatidylinositol (PI), **(D)** phosphatidic acid (PA), **(E)** phosphatidylcholine (PC), **(F)** phosphatidylserine (PS), and **(G)** phosphatidylglycerol (PG) lipid species. The color bars represent the ratio of DN/NC in each lipid species, with the relative abundance of the normal group fixed as a value of 1. Only statistically significant changes are shown. Data are presented as means ± SEM, *n* = 6 per group; *P < 0.05, **P < 0.01, ***P < 0.001.

As shown in Figure 4A, the comprehensive alterations in the relative abundance of phospholipids were analyzed. The total class content of PE, PA, and PS exhibited uniform reduction. Ratio heat plots were generated using the normalized intensity of the DN group compared with that of the NC group, in which the relative abundance within the NC group was assigned a fixed value of 1 (Figures 4B–H). Lipids with side chains of 36:1 and 36:2, including PE, PA, PI, PS, and PC, showed a heterogeneous increase in DN kidneys. Although no gross perturbations in PG or PI content were observed, those of PG 32:1 and PG 34:1 decreased significantly. Furthermore, PI with more than four double bonds showed a significant decrease in the DN kidney cortex. Taken together, the results show that remodeling of the kidney cortex during early-stage DN was characterized by a reduction in phospholipids, except in those with side chains of 36:1 or 36:2.

### Diverse Alterations in Lyso-Phospholipids During Early-Stage Diabetic Nephropathy

**Figure 5:**
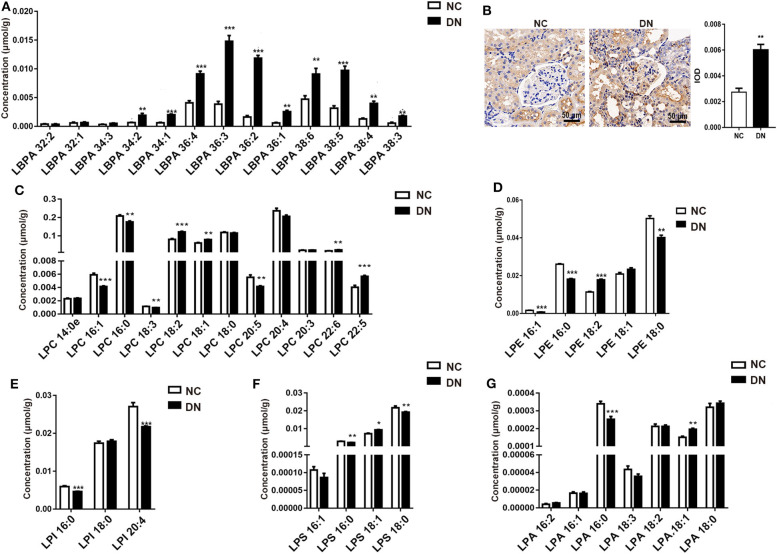
Diabetic nephropathy differentially affected the abundance of lyso-phospholipids. After diabetic nephropathy (DN) was induced by feeding high-fat diet (HFD) in combination with injection of low-dose streptozotocin (STZ) for 8 weeks, **(A)** the content of specific species of lyso-bisphosphatidic acid (LBPA) within the kidney cortex was analyzed; *n* = 6 per group. **(B)** Immunohistochemical staining of CD68, at 400× magnification, and quantitative analysis of positivity in kidney glomeruli are shown. Five glomeruli were assessed for each slide; *n* = 3 per group. Content of specific species of **(C)** lyso-phosphatidylcholine (LPC), **(D)** lyso-phosphatidylethanolamine (LPE), **(E)** (LPI), **(F)** LPS, and **(G)** LPA in the kidney cortex was analyzed; *n* = 6 per group. Data are presented as means ± SEM; *P < 0.05, **P < 0.01, ***P < 0.001.

LBPA plays a crucial role in macrophage biology and function (14). Here, we observed a drastic alteration in LBPA in DN rats. As shown in Figure 5A, most LBPA molecules contained long-chains (C34, C36, and C38). Unsaturated acyl-chains, especially LBPA (36:2), increased by 2- to 4-fold in the DN group, with a 7.3-fold increase observed relative to the normal group. The significant increase in LBPA indicated the activation of macrophages in kidneys in the DN group, which was confirmed by the detection of detection of higher levels of the macrophage marker CD68 in DN glomeruli (Figure 5B). We examined other lyso-phospholipids, including LPC, LPE, LPI (lyso-phosphatidylinositols), LPS, and LPA (lyso-phosphatidic acids). Among them, the levels of LPC with side chains of LPC(16:1), LPC(18:2), LPC(22:5), and LPC(22:6) significantly increased, whereas LPC with side chains of LPC(16:0), LPC(18:3), and LPC(20:5) decreased in the DN kidney cortex (Figure 5C). Interestingly, unsaturated LPEs, including LPE(16:0) and LPE(18:0), were reduced in the DN group, whereas LPE(18:2) showed an increase of 50% in comparison with the normal group (Figure 5D). All LPI species decreased in the DN group (Figure 5E). The composition of LPS showed the same pattern as that of LPE, in that LPS containing unsaturated fatty acid chains, such as LPS(16:0) and LPS (18:0), was reduced, whereas LPS(18:1) was significantly increased (Figure 5F). As shown in Figure 5G, LPA(16:0) decreased by 25%, whereas LPA(18:1) increased by 30% in the DN kidney cortex. Collectively, LBPA exhibited a homogeneous increase, whereas other lyso-phospholipids showed variable changes. The precise pathological relationships between these various lipid species during DN require further investigation.

### Comprehensive Changes in Sphingolipids During Early-Stage Diabetic Nephropathy

**Figure 6:**
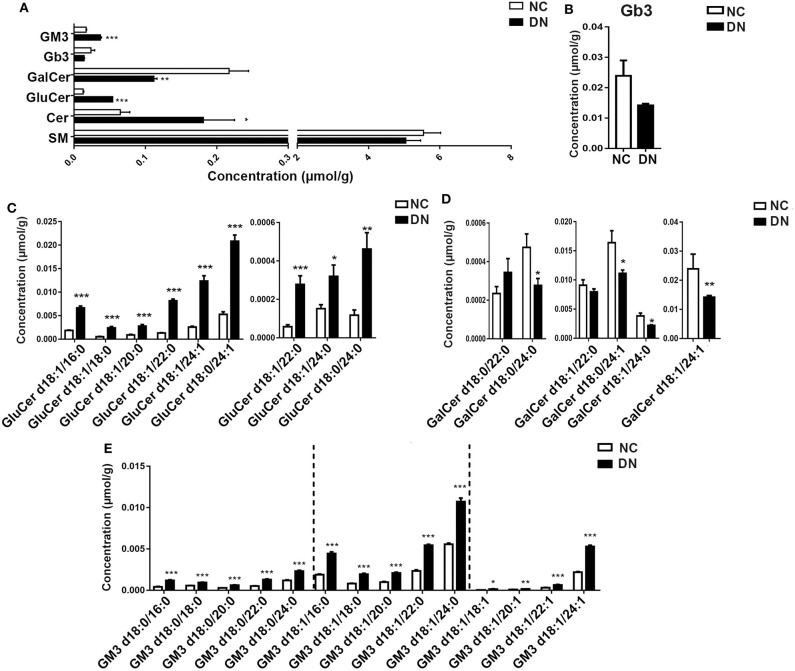
Sphingolipids were significantly altered in the diabetic kidney cortex. Total content of sphingolipid classes **(A)** and specific species of Gb3 **(B)**, glucosylceramide (GluCer) **(C)**, galactosylceramide (GalCer) **(D)**, and monosialo-dihexosyl gangliosides (GM3) **(E)** were analyzed. Data are presented as means ± SEM, *n* = 6 per group; *P < 0.05, **P < 0.01, ***P < 0.001.

Sphingolipids, which are enriched in the kidney cortex, play important roles in the regulation of cellular function (5). In the current study, we analyzed several classes of sphingolipids and their species. As shown in Figure 6A, most sphingolipids were increased in DN rats without alteration of total sphingomyelin. Globotriaosylceramide (Gb3) showed a decreasing tendency in DN, although the decrease was not significant (Figure 6B). Although the total Cer significantly increased, the changes in ceramide derivatives were not consistent. GluCer markedly decreased, whereas GalCer and monosialo-dihexosyl gangliosides (GM3) increased significantly. We then examined the alteration of species in detail. Results showed that all species of GluCer greatly increased, especially GluCer d18:1/22:0, which increased by 6.25-fold in the DN kidney cortex (Figure 6C). Interestingly, in the DN group, most GalCer species decreased by 20–40% compared with those in the normal group, except for GalCer d18:0/22:0 (Figure 6D). GM3, the most abundant ganglioside, is characterized by the presence of sialic acid groups linked to the ceramide skeleton structure. As shown in Figure 6E, compared with those in the NC group, all GM3 species were significantly increased in DN rats; in particular, the most abundant species of GM3, which contained one unsaturated bond, was intensely elevated. Specific GM3 species were identified in three classes on the basis of their number of unsaturated bonds; among these, GM3 with a side chain of 24 carbons was predominant. Moreover, differences in GM3 between the NC and DN groups were positively correlated with chain length, except in GM3 d18:1/18:1, which was decreased by 4.66-fold in the DN kidney cortex. Collectively, marked alterations were observed in sphingolipids in the early-stage DN model, as evidenced by a homogenous increase in GM3 and diverse changes in GluCer and GalCer. Such changes may serve as potential biomarkers; however, further studies are required to verify this.

## Discussion

Abnormal lipid accumulation induces detrimental changes to renal lipid metabolism that promotes insulin resistance, oxidative stress, and endoplasmic reticulum (ER) stress. These changes ultimately result in deterioration of renal structure and function (15). The most detrimental effect of abnormal lipid accumulation is damage to the glomerular filtration barrier (GFB) as a result of degradation of the glycocalyx, which induces podocyte death and mesangial cell instability (16). These injuries lead to increased GFB permeability, which is a hallmark of, and central event in, DN progression. Lipotoxicity promotes the development of early-stage DN prior to epithelial–mesenchymal transition (EMT) (17). Therefore, comprehensive lipidome analysis is crucial to the identification of potential biomarkers of DN and elucidation of the mechanistic basis of DN. Herein, we applied a high-coverage, targeted lipidomic approach to analyze the lipidome of the kidney cortex in early-stage DN rats, and we identified a lipid profile comprising 437 lipid species from 25 classes. Glycerolipids, sterol lipids, phospholipids, and sphingolipids were additionally identified in this study.

Neutral lipids are closely associated with DN progression. In this study, neutral lipids, such as TAG, DAG, FFA, Cho, and CE, were found to be major components of the kidney lipidome. The observed increase in blood lipids was accompanied by a homogeneous increase in glyceride lipids in the DN kidney cortex. Podocytes are the main site of excess lipid accumulation in DN (7); however, these cells are particularly sensitive to lipid accumulation and undergo induction of insulin resistance or cell death readily as a result of this phenomenon (18). In this study, a significant increase in FFA chain length and degree of unsaturation was observed in the DN kidney cortex. Elevated FFAs not only induced aggravated dyslipidemia and diabetes by increasing plasma TAG but also induced podocyte injury and exacerbated proteinuria by stimulating aberrant micropinocytosis by podocytes (19). Further, fatty acid metabolites, DAG, and ceramides were also elevated. Excessive accumulation of FFA in non-adipose organs results in toxicity and death of tubular epithelial cells in the diabetic kidney (20). A notable finding in this study was that highly unsaturated TAG species markedly accumulated in the cortex of DN kidneys, indicative of susceptibility to lipid peroxidation. Downregulation of fatty acid β-oxidation pathways as well as TAG hydrolase, including PPAR-α, carnitine palmitoyltransferase 1 (CPT1), acyl-CoA oxidase (ACO), and L-FABP, has been strongly linked to glomerular hyperfiltration and inflammation as evidenced by studies of biopsies from DM patients (7). Such TAG species might therefore be potentially relevant to inflammatory processes in DN progression. In a previous study, CE with 23C containing two to four unsaturated bonds was positively related to diabetes (21), and CE with 20C was positively related to T2DM development in women with previous gestational DM (22). Interestingly, in this study, CE with 20C containing two to four unsaturated bonds dramatically increased by 15- to 23-fold in the kidney cortex of early-stage DN model rats. Further, an increase in the side chains of linoleic acid (18:2) was observed in TAG, DAG, FFA, and CE, indicating that linoleic acid plays an important role in DN progression. Wang et al. confirmed that linoleic acid served as a potential indicator of the occurrence and development of DN (23). Furthermore, we observed a dramatic increase in CE containing linoleic acid (18:2) in the DN kidney cortex, with a 28.3-fold increase relative to normal rats. However, further investigation is necessary to establish whether linoleic acid or glycerides can serve as positive indicators for DN progression.

Although phospholipids constitute the minority of the kidney cortex lipidome, they carry out many crucial biological functions such as the maintenance of cellular membrane stability and regulation of cell signaling (24). In this study, significantly altered phospholipid metabolism was characterized by remodeling of polyunsaturated PI as well as PE. In a previous study, phospholipidomic analysis of human plasma demonstrated that PI C18:0/22:6 could be used to discriminate healthy individuals from T2DM or DN patients (8). Consistent with this previous finding, we observed significantly decreased PI 40:6 as well as PI 38:6 in the early-stage DN rat model in this study. Amadori-glycated PE formation triggers lipid peroxidation and may serve as a pathogenic factor during DN (25, 26). However, using matrix-assisted laser desorption ionization imaging mass spectrometry (MALDI IMS), Grove et al. found that Amadori-PEs played only a minor role in DN pathogenesis (24). Here, we observed a homogeneous decline in PE with or without plasmalogen bonds. Decreased PE in the DN cortex has also been observed in progressive DN induced by uninephrectomy with a single intraperitoneal injection of STZ (9). These observations indicate that PE plays an important role in the maintenance of renal function. Although the total amount of PC barely changed, there was a significant decrease in total PE within the DN kidney cortex. Hence, the ratio of PC/PE increased drastically, which has been reported to contribute to ER stress (27). The conversion of PE to PC is catalyzed by phosphatidylethanolamine *N*-methyltransferase (Pemt). Glomerular hypertrophy and albuminuria have been shown to be significantly attenuated in Pemt-deficient mice as a result of a decrease in the ratio of PC/PE and alleviation of ER stress (28). Interestingly, although most phospholipids declined, a specific side chain, 36:1, was increased in almost every phospholipid, including PE, PI, PA, PG PC, and PS. In addition, PC(36:1) has been reported to be independently associated with increased risk for T2DM (29). However, the 36:1 side chain of phospholipids has been rarely reported. The potential for this side chain to serve as a specific biomarker for DN, that is, to distinguish DM from DN, requires further study.

LBPA, also known as bis(monoacylglycerol)phosphate, is crucial for macrophage biology and activity because of its unique role in lysosomal function and storage (14). In this study, elevated LBPA levels were observed in the DN kidney cortex; most of the increased LBPA contained long-chain unsaturated fatty acids, indicating intrinsic regulation of macrophages in DN. The upregulation of CD68 protein levels is consistent with enhanced macrophage infiltration into diabetic glomeruli. Previous studies have revealed that LPA and LPC participate in the pathogenesis of chronic kidney disease through receptor-dependent promotion of proliferation, inflammation, and fibrosis within the kidney (30, 31). Although saturated LPC is usually associated with pro-inflammatory conditions, polyunsaturated LPC is considered to prevent the inflammatory response. In this study, we observed significant accumulation of LPC(18:1), LPC(18:2), LPC(22:5), LPC(22:6), and LPA(18:1) in the kidneys of DN rats. These results are partially consistent with Saulnier-Blache's observation of the urinary lipidome in T2DM patients, in which LPC(18:0) and LPC(18:1) were significantly elevated, along with macro-albuminuria and mildly reduced estimated glomerular filtration rate (eGFR) (32). Although LPA and LPC are known to promote renal inflammation and tubulo-interstitial fibrosis, some species of LPA and LPC, such as LPC(16:0) and LPC(16:1), are rarely reported in the normal kidney but significantly decreased in the kidneys of DN rats. However, the mechanisms by which these lyso-phospholipids influence the progression of DN are unknown.

It has been reported that sphingolipid accumulation contributes to the development of diabetic kidney disease (33). Consistent with this report, we observed that most sphingolipids were dramatically elevated in the kidney cortex of early-stage DN rats. In the current study, we examined ceramides and their derivatives, including GluCer, GalCer, SM, GM3, and Gb3. Consistent with the results of Liu et al. in a type 1 diabetic rat model (33), most ceramides were greatly increased in the present DN rat model. However, in a type 2 db/db mouse model of DN, ceramides were decreased in the kidney cortex and increased in the plasma (6). One possible explanation for this discrepancy related to DN stage; in the type 2 DN rat model used in this study, kidney cortex tissue was obtained shortly after onset, during the early stages of DN. Sas et al. examined the renal cortex of diabetic mice 24 weeks after the onset of severe renal fibrosis (6). Partial confirmation of this hypothesis was provided by the work of Geoffroy, who demonstrated that the activity and expression of neutral ceramidase was elevated in the diabetic glomeruli 4 days after induction of diabetes but decreased after 28 days (34).

GluCer, which is one of the simplest subclasses of glycosphingolipids (GSLs), is generated from ceramide UDP-Glc:ceramide glucosyltransferase (35). Increasing evidence has been reported for the key role of GSLs in insulin resistance (36). In a previous study, GluCer was found to be increased in early-stage DN (9 weeks) and to increase further with advanced DN (17 weeks), with elevated GSLs mediating renal mesangial hypertrophy (37). However, this previous study did not distinguish individual species of GSLs. Applying advanced lipidomics, we distinguished between the individual species of GSLs on the basis of fatty acyl groups and, unexpectedly, found that the levels of most GalCer species decreased in the DN kidney cortex. Ceramides with the α-anomeric linkage of a galactose sugar are lipid antigens, such as α-GalCer, which strongly activates natural killer (NK) T-cells (38) and inhibits the development of T-cell-mediated autoimmune type 1 diabetes (30, 31). Furthermore, Uchida et al. reported that administration of α-GalCer caused damage to renal vascular endothelial cells as well as tubular epithelial cells and ultimately led to acute kidney injury (39). However, few reports have demonstrated a relationship between GalCer and the progression of DN. Because inflammation is key to the onset and progression of DN, further investigation is required to determine how decreased GalCer in DN disrupts the balance of inflammatory mediators and the mechanisms underlying the resulting toxic effects against glomerular endothelial cells.

GM3, the most abundant ganglioside and predominant lipid in the kidney, is involved in a variety of cellular functions including signal transduction, proliferation, differentiation, and apoptosis (40, 41). Interestingly, our results showed that GM3, especially GM3 containing one unsaturated bond, increased 2- to 4-fold in the DN kidney cortex. In contrast, Kwak et al. observed a decrease in GM3 in STZ-induced diabetic rat glomeruli and the consequent loss of a charge-selective filtration barrier in the renal glomeruli (42). Other studies have shown that GM3 compromises cell regeneration of the GFB through VEGF and AKT pathways (43, 44). Further, GM3 is an important component of lipid rafts (45), which are key to renal SGLT2 and Na/K/Cl co-transporters (46). Therefore, increased GM3 may in turn increase tubular reabsorption and efferent arterial hydrostatic pressure by upregulating Na^+^-glucose co-transporter activity, resulting in the decreased glomerular filtration. Furthermore, podocyte function depends on the integrity of lipid rafts, which are largely composed of sphingolipids, including gangliosides (47). In the current study, not all GM3s showed a marked increase; only GM3 containing the oleic acid (18:1) side chain, including GM3 d18:1/24:0, GM3 d18:1/22:0, and GM3 d18:1/24:1, was elevated. Although GM3 has been reported to exert deleterious effects on renal function and structure, few studies have sought to identify the species that are related to the progression of DN. The results from the present lipidomic analysis of GM3 in the kidneys of our DN rat model may offer new insights into the mechanistic basis of DN.

In conclusion, the comparison of the lipidome of the kidney cortex of normal and diabetic rats revealed a distinguishing signature for the DN kidney lipidome, which is characterized by changes in side chain composition and unsaturated bonds. Neutral lipids comprised the majority of the DN kidney lipidome, with most exhibiting a higher degree of unsaturation and side chains of linoleic acid, which may serve as potential markers for DN. Substantial changes were observed in the least abundant lipid classes, especially phospholipids and sphingolipids such as ceramide and GM3. These may exert deleterious effects on the GFB during early-stage DN. The present results provide a detailed overview of the lipidome of the kidney cortex in DN; additionally, the findings offer new insights into the mechanistic basis of DN and reveal pathologically relevant lipid species. However, further investigation is required to identify specific biomarkers for the timely diagnosis of DN.

## Data Availability Statement

The datasets generated for this study are available on request to the corresponding author.

## Ethics Statement

The animal study was reviewed and approved by Animal care committee of Institute of Materia Medica, Chinese Academy of Medical Sciences.

## Author Contributions

BH: conceptualization. PH, PM, and XinY: investigation. SL and GS: methodology. XiuY, LZ, GQ, and GD: supervision. CX: visualization. BH: Writing-original draft. GQ: writing-review and editing. All authors contributed to the article and approved the submitted version.

## Conflict of Interest

The authors declare that the research was conducted in the absence of any commercial or financial relationships that could be construed as a potential conflict of interest.
