# Supplementary material for: Comprehensive Lipidome Profiling of the Kidney in Early-Stage Diabetic Nephropathy
Source: Front Endocrinol (Lausanne). 2020 Jun 19;11:359. doi: 10.3389/fendo.2020.00359 (PMC7325916; doi:10.3389/fendo.2020.00359)
Supplement: Supplementary file 1 [file Table_1.pdf]

Table 1 lipidomic results of diabetic kidney cortex and normal kidney cortex

Concentration (μmol/g)	NC						DN					
	NC1	NC2	NC3	NC4	NC5	NC6	DN1	DN2	DN3	DN4	DN5	DN6
DAG32:2(16:1/16:1)	0.02377	0.02441	0.02528	0.02125	0.02774	0.02717	0.02315	0.02311	0.02283	0.02383	0.02343	0.01901
DAG32:1(16:1/16:0)	0.01851	0.02147	0.02047	0.01891	0.02135	0.02328	0.01837	0.01780	0.01764	0.02122	0.01978	0.02112
DAG34:2(16:0/18:2)	0.11017	0.12790	0.10328	0.09389	0.10710	0.12703	0.13672	0.13693	0.14823	0.14120	0.12801	0.12617
DAG34:1(16:0/18:1)	0.09315	0.11403	0.10500	0.10246	0.11506	0.11733	0.15324	0.16905	0.17916	0.17285	0.16004	0.16107
DAG36:4(18:2/18:2)	0.34081	0.42052	0.35377	0.35098	0.38015	0.38769	0.35604	0.38514	0.41412	0.39004	0.37414	0.38206
DAG36:3(18:2/18:1)	0.10126	0.11016	0.09036	0.08933	0.10288	0.10284	0.17280	0.21077	0.18568	0.20604	0.19820	0.19253
DAG36:2(18:1/18:1)	0.48070	0.55042	0.49205	0.47490	0.51620	0.56724	0.60676	0.65681	0.68316	0.68298	0.63468	0.63961
DAG36:2(18:2/18:0)	0.11177	0.13764	0.12250	0.11291	0.13411	0.14307	0.19888	0.22059	0.22518	0.22892	0.21625	0.22565
DAG36:1(18:1/18:0)	0.15132	0.18752	0.16139	0.15129	0.17693	0.18459	0.25855	0.27063	0.28498	0.28974	0.25796	0.26323
DAG38:6(18:1/20:5)	0.02617	0.02667	0.02528	0.02547	0.02690	0.02717	0.02250	0.02427	0.02779	0.02703	0.02644	0.02579
DAG38:4(18:0/20:4)	0.16195	0.20352	0.17191	0.17465	0.19731	0.19016	0.11009	0.11844	0.12588	0.12460	0.11533	0.11549
DAG40:6(18:0/22:6)	0.26344	0.37131	0.37939	0.32161	0.26582	0.26557	0.32854	0.23122	0.36785	0.30918	0.34007	0.29813
DAG40:5(18:0/22:5)	0.05406	0.08336	0.08270	0.06586	0.05656	0.06610	0.06238	0.06251	0.07647	0.06757	0.06718	0.06492
TAG48:4(18:2)	0.00122	0.00122	0.00043	0.00106	0.00184	0.00175	0.00113	0.00153	0.00140	0.00154	0.00199	0.00063
TAG48:4(16:1)	0.00329	0.00261	0.00268	0.00274	0.00289	0.00341	0.00253	0.00322	0.00428	0.00279	0.00306	0.00325
TAG48:3(18:2)	0.00107	0.00171	0.00156	0.00186	0.00281	0.00070	0.00122	0.00225	0.00166	0.00154	0.00252	0.00111
TAG48:3(18:1)	0.00405	0.00350	0.00502	0.00495	0.00438	0.00446	0.00497	0.00451	0.00446	0.00463	0.00405	0.00467
TAG48:3(16:1)	0.00604	0.00570	0.00476	0.00628	0.00605	0.00568	0.00785	0.00499	0.00638	0.00529	0.00527	0.00364
TAG48:3(16:0)	0.00199	0.00163	0.00156	0.00247	0.00228	0.00192	0.00323	0.00330	0.00367	0.00279	0.00267	0.00285
TAG48:2(18:2)	0.00252	0.00326	0.00398	0.00212	0.00272	0.00341	0.00890	0.00821	0.00664	0.00618	0.00672	0.00919

TAG48:2(18:1)	0.00811	0.01067	0.00649	0.00955	0.00815	0.00971	0.01030	0.00861	0.01014	0.00632	0.00825	0.00737
TAG48:2(16:1)	0.00199	0.00204	0.00268	0.00194	0.00210	0.00201	0.00445	0.00330	0.00280	0.00294	0.00328	0.00325
TAG48:2(16:0)	0.00382	0.00423	0.00433	0.00468	0.00351	0.00359	0.00750	0.00813	0.01005	0.00588	0.00894	0.00982
TAG48:1(18:0)	0.00092	0.00106	0.00216	0.00062	0.00140	0.00122	0.00236	0.00137	0.00227	0.00081	0.00244	0.00111
TAG48:1(18:1)	0.00903	0.00798	0.01013	0.00778	0.00938	0.00726	0.01466	0.01522	0.01905	0.01243	0.01497	0.01568
TAG48:1(16:0)	0.00642	0.00635	0.00598	0.00460	0.00903	0.00787	0.01632	0.01964	0.01818	0.01500	0.01581	0.01442
TAG48:1(16:1)	0.00413	0.00285	0.00407	0.00442	0.00386	0.00437	0.00620	0.00668	0.00795	0.00640	0.00657	0.00642
TAG50:4(18:2)	0.00092	0.00204	0.00251	0.00150	0.00175	0.00184	0.00157	0.00201	0.00315	0.00110	0.00191	0.00356
TAG50:4(16:2)	0.00153	0.00090	0.00121	0.00080	0.00096	0.00114	0.00061	0.00113	0.00149	0.00103	0.00092	0.00055
TAG50:3(18:2)	0.00352	0.00326	0.00407	0.00265	0.00377	0.00472	0.00611	0.00564	0.00481	0.00588	0.00688	0.00618
TAG50:3(18:1)	0.00382	0.00285	0.00546	0.00433	0.00447	0.00647	0.00777	0.00419	0.00551	0.00581	0.00497	0.00737
TAG50:3(16:0)	0.00314	0.00456	0.00537	0.00574	0.00377	0.00446	0.00934	0.00934	0.00778	0.00816	0.00802	0.00713
TAG50:3(16:1)	0.00421	0.00342	0.00433	0.00486	0.00412	0.00376	0.00593	0.00789	0.00551	0.00559	0.00871	0.00713
TAG50:3(16:2)	0.00061	0.00065	0.00052	0.00088	0.00070	0.00087	0.00166	0.00113	0.00149	0.00110	0.00191	0.00143
TAG50:2(18:2)	0.01476	0.01889	0.02390	0.02236	0.02200	0.02230	0.05680	0.06553	0.07360	0.06176	0.06050	0.06519
TAG50:2(18:1)	0.00627	0.00879	0.00805	0.00893	0.00798	0.00813	0.01213	0.01361	0.01512	0.01272	0.01008	0.01363
TAG50:2(16:0)	0.03258	0.03445	0.03403	0.03677	0.03428	0.03778	0.11282	0.10135	0.11730	0.11698	0.10947	0.11542
TAG50:2(16:1)	0.00314	0.00342	0.00520	0.00362	0.00508	0.00455	0.00855	0.01095	0.00944	0.00691	0.01062	0.00729
TAG50:2(16:2)	0.00038	0.00041	0.00035	0.00027	0.00035	0.00052	0.00096	0.00137	0.00157	0.00088	0.00046	0.00079
TAG50:1(16:1)	0.00176	0.00179	0.00069	0.00159	0.00114	0.00079	0.00358	0.00443	0.00358	0.00324	0.00367	0.00412
TAG50:1(16:0)	0.02256	0.03787	0.03481	0.04074	0.03691	0.03603	0.16587	0.15457	0.15934	0.13353	0.15417	0.15764
TAG50:1(18:0)	0.00176	0.00195	0.00182	0.00221	0.00219	0.00140	0.00558	0.00386	0.00516	0.00500	0.00413	0.00594
TAG50:1(18:1)	0.02187	0.02508	0.02511	0.02908	0.02831	0.02799	0.10060	0.10264	0.11258	0.09110	0.09794	0.10052
TAG52:4(16:0)	0.00811	0.00912	0.01117	0.00857	0.00929	0.01268	0.01579	0.01610	0.01827	0.01353	0.01566	0.01465

TAG52:4(16:1)	0.00184	0.00187	0.00268	0.00380	0.00202	0.00227	0.00227	0.00290	0.00332	0.00441	0.00313	0.00293
TAG52:4(16:2)	0.00099	0.00049	0.00113	0.00133	0.00079	0.00105	0.00113	0.00064	0.00052	0.00059	0.00099	0.00087
TAG52:4(18:1)	0.00543	0.00497	0.00779	0.00619	0.00587	0.00490	0.00768	0.00716	0.00656	0.00654	0.00474	0.00610
TAG52:4(18:2)	0.00964	0.01409	0.01576	0.01432	0.01306	0.01084	0.02940	0.02697	0.02771	0.02515	0.02613	0.02646
TAG52:4(18:3)	0.00252	0.00130	0.00199	0.00362	0.00175	0.00166	0.00515	0.00362	0.00420	0.00346	0.00390	0.00261
TAG52:3(16:0)	0.01300	0.01531	0.01749	0.01865	0.01929	0.01565	0.04275	0.04226	0.04799	0.03801	0.04729	0.04159
TAG52:3(16:1)	0.00283	0.00171	0.00398	0.00186	0.00237	0.00350	0.00489	0.00467	0.00428	0.00419	0.00428	0.00436
TAG52:3(16:2)	0.00069	0.00122	0.00052	0.00088	0.00123	0.00105	0.00070	0.00072	0.00087	0.00074	0.00115	0.00143
TAG52:3(18:1)	0.01706	0.01906	0.02026	0.02112	0.01999	0.02108	0.05148	0.04814	0.05349	0.04485	0.05531	0.05054
TAG52:3(18:2)	0.01361	0.01555	0.01715	0.01723	0.01341	0.01618	0.04546	0.04645	0.04598	0.04625	0.04782	0.04690
TAG52:2(18:2)	0.00314	0.00554	0.00675	0.00566	0.00508	0.00472	0.03193	0.02729	0.03339	0.02456	0.02544	0.03295
TAG52:2(18:1)	0.02126	0.02435	0.02875	0.02793	0.02709	0.02580	0.09197	0.08606	0.09519	0.07169	0.09045	0.08389
TAG52:2(16:0)	0.01117	0.01344	0.01784	0.01944	0.01508	0.01408	0.06265	0.05828	0.06695	0.05897	0.05233	0.06179
TAG52:2(16:1)	0.00145	0.00114	0.00173	0.00062	0.00114	0.00184	0.00148	0.00266	0.00411	0.00235	0.00252	0.00206
TAG52:1(18:1)	0.00895	0.01018	0.01325	0.01343	0.01254	0.01006	0.04886	0.04436	0.04528	0.04676	0.04683	0.04119
TAG52:1(16:1)	0.00145	0.00090	0.00191	0.00141	0.00044	0.00122	0.00148	0.00137	0.00201	0.00044	0.00290	0.00111
TAG54:8(20:4)	0.00088	0.00094	0.00069	0.00075	0.00074	0.00108	0.00055	0.00088	0.00159	0.00084	0.00062	0.00036
TAG54:8(18:3)	0.00088	0.00102	0.00145	0.00097	0.00133	0.00100	0.00195	0.00081	0.00106	0.00126	0.00180	0.00094
TAG54:8(22:6)	0.00102	0.00071	0.00107	0.00149	0.00169	0.00091	0.00187	0.00168	0.00061	0.00147	0.00111	0.00144
TAG54:7(22:6)	0.00129	0.00189	0.00214	0.00179	0.00184	0.00174	0.00179	0.00146	0.00151	0.00147	0.00076	0.00216
TAG54:7(20:4)	0.00211	0.00071	0.00214	0.00194	0.00059	0.00141	0.00148	0.00110	0.00144	0.00049	0.00090	0.00079
TAG54:7(18:2)	0.00109	0.00134	0.00161	0.00246	0.00125	0.00108	0.00125	0.00220	0.00348	0.00161	0.00131	0.00281
TAG54:7(18:3)	0.00122	0.00220	0.00253	0.00179	0.00324	0.00216	0.00234	0.00220	0.00265	0.00238	0.00262	0.00209
TAG54:6(20:4)	0.00252	0.00228	0.00276	0.00381	0.00354	0.00274	0.00444	0.00432	0.00234	0.00328	0.00380	0.00425

TAG54:6(18:2)	0.00768	0.01022	0.01263	0.01165	0.00928	0.01294	0.01247	0.01018	0.01119	0.01272	0.01078	0.01383
TAG54:6(18:3)	0.00279	0.00354	0.00199	0.00261	0.00243	0.00365	0.00405	0.00359	0.00325	0.00370	0.00380	0.00317
TAG54:5(20:4)	0.00252	0.00267	0.00230	0.00187	0.00273	0.00315	0.00499	0.00498	0.00575	0.00356	0.00504	0.00511
TAG54:5(18:1)	0.01509	0.01878	0.01951	0.02053	0.01812	0.01650	0.02657	0.02819	0.02398	0.02418	0.02127	0.02600
TAG54:5(18:2)	0.01360	0.02232	0.02495	0.01986	0.01613	0.02189	0.02759	0.02943	0.02700	0.02775	0.02397	0.03003
TAG54:5(18:3)	0.00347	0.00385	0.00490	0.00336	0.00317	0.00340	0.00390	0.00505	0.00484	0.00349	0.00518	0.00418
TAG54:5(16:0)	0.00122	0.00189	0.00245	0.00202	0.00206	0.00282	0.00335	0.00381	0.00325	0.00294	0.00311	0.00281
TAG54:5(16:1)	0.00109	0.00063	0.00084	0.00090	0.00052	0.00124	0.00148	0.00124	0.00091	0.00049	0.00111	0.00086
TAG54:5(16:2)	0.00034	0.00086	0.00077	0.00075	0.00059	0.00017	0.00094	0.00022	0.00038	0.00035	0.00035	0.00043
TAG54:4(18:3)	0.00163	0.00157	0.00138	0.00164	0.00265	0.00232	0.00390	0.00308	0.00219	0.00294	0.00283	0.00295
TAG54:4(18:2)	0.02801	0.03269	0.03704	0.03434	0.03381	0.04155	0.07552	0.08406	0.07374	0.07408	0.07487	0.06374
TAG54:4(18:1)	0.06227	0.06924	0.07033	0.06458	0.06623	0.06618	0.10949	0.09921	0.09901	0.09896	0.09912	0.09377
TAG54:4(18:0)	0.00483	0.00456	0.00597	0.00605	0.00545	0.00672	0.01691	0.01889	0.01936	0.01810	0.01844	0.01764
TAG54:4(16:0)	0.00292	0.00173	0.00276	0.00351	0.00287	0.00224	0.00694	0.00483	0.00499	0.00468	0.00504	0.00497
TAG54:4(16:1)	0.00095	0.00086	0.00069	0.00030	0.00066	0.00041	0.00039	0.00051	0.00091	0.00084	0.00090	0.00086
TAG54:4(16:2)	0.00020	0.00071	0.00077	0.00030	0.00007	0.00041	0.00070	0.00066	0.00015	0.00049	0.00048	0.00058
TAG54:3(16:0)	0.00184	0.00189	0.00145	0.00157	0.00162	0.00199	0.00514	0.00637	0.00355	0.00531	0.00435	0.00634
TAG54:3(16:1)	0.00054	0.00024	0.00031	0.00022	0.00088	0.00166	0.00055	0.00037	0.00023	0.00091	0.00062	0.00094
TAG54:3(18:1)	0.11325	0.13368	0.14273	0.12758	0.12708	0.14812	0.22616	0.19726	0.20717	0.20994	0.18138	0.20871
TAG54:3(18:2)	0.01013	0.01312	0.01515	0.01120	0.01392	0.01377	0.05697	0.04481	0.04047	0.04396	0.05028	0.04861
TAG54:2(18:2)	0.00211	0.00393	0.00360	0.00299	0.00332	0.00307	0.01644	0.01772	0.01513	0.01817	0.01817	0.01837
TAG54:2(18:1)	0.04371	0.05156	0.05319	0.05181	0.04855	0.05490	0.09141	0.09841	0.10362	0.10245	0.09449	0.10140
TAG54:2(18:0)	0.01088	0.01548	0.01531	0.01389	0.01216	0.01841	0.06788	0.06451	0.06429	0.06513	0.07163	0.07130
TAG54:1(18:0)	0.00755	0.00865	0.01048	0.00747	0.00832	0.00846	0.03577	0.03266	0.03146	0.03201	0.03129	0.03399

TAG54:1(18:1)	0.00931	0.00904	0.01064	0.00844	0.00958	0.01111	0.02556	0.02541	0.02360	0.02621	0.02348	0.02564
TAG56:8(22:6)	0.00102	0.00244	0.00099	0.00216	0.00103	0.00274	0.00413	0.00322	0.00318	0.00384	0.00387	0.00202
TAG56:8(22:5)	0.00095	0.00008	0.00084	0.00105	0.00059	0.00033	0.00047	0.00073	0.00076	0.00070	0.00083	0.00101
TAG56:8(18:1)	0.00843	0.01053	0.01071	0.00948	0.01083	0.01045	0.01068	0.01018	0.01021	0.01244	0.01112	0.01095
TAG56:8(18:2)	0.00163	0.00118	0.00184	0.00090	0.00059	0.00182	0.00288	0.00205	0.00212	0.00266	0.00269	0.00266
TAG56:7(22:6)	0.00265	0.00346	0.00184	0.00157	0.00265	0.00307	0.00592	0.00527	0.00590	0.00538	0.00449	0.00699
TAG56:7(22:5)	0.00163	0.00047	0.00046	0.00127	0.00125	0.00182	0.00101	0.00095	0.00113	0.00203	0.00117	0.00230
TAG56:7(20:2)	0.00020	0.00047	0.00077	0.00045	0.00059	0.00066	0.00000	0.00029	0.00053	0.00063	0.00055	0.00029
TAG56:7(20:4)	0.00143	0.00189	0.00145	0.00090	0.00140	0.00149	0.00382	0.00359	0.00408	0.00287	0.00380	0.00411
TAG56:7(20:5)	0.00088	0.00047	0.00084	0.00082	0.00118	0.00066	0.00070	0.00146	0.00113	0.00182	0.00041	0.00166
TAG56:7(18:2)	0.00102	0.00157	0.00145	0.00097	0.00133	0.00124	0.00242	0.00381	0.00303	0.00189	0.00200	0.00216
TAG56:7(18:3)	0.00027	0.00118	0.00122	0.00127	0.00111	0.00191	0.00062	0.00110	0.00076	0.00147	0.00048	0.00108
TAG56:6(22:5)	0.00122	0.00181	0.00207	0.00067	0.00133	0.00249	0.00234	0.00256	0.00166	0.00231	0.00249	0.00166
TAG56:6(22:4)	0.00143	0.00157	0.00138	0.00119	0.00081	0.00216	0.00070	0.00161	0.00144	0.00196	0.00138	0.00122
TAG56:6(20:1)	0.00061	0.00063	0.00069	0.00075	0.00044	0.00075	0.00117	0.00059	0.00045	0.00049	0.00035	0.00065
TAG56:6(20:2)	0.00027	0.00079	0.00084	0.00067	0.00015	0.00008	0.00101	0.00088	0.00045	0.00112	0.00104	0.00050
TAG56:6(20:3)	0.00075	0.00204	0.00168	0.00134	0.00111	0.00124	0.00156	0.00227	0.00310	0.00328	0.00290	0.00288
TAG56:6(20:4)	0.00265	0.00212	0.00153	0.00209	0.00155	0.00224	0.00686	0.00864	0.00529	0.00650	0.00739	0.00583
TAG56:6(18:2)	0.00122	0.00086	0.00230	0.00112	0.00133	0.00232	0.00397	0.00308	0.00280	0.00363	0.00311	0.00411
TAG56:6(18:3)	0.00082	0.00173	0.00092	0.00142	0.00044	0.00083	0.00226	0.00168	0.00121	0.00175	0.00152	0.00122
TAG56:5(22:4)	0.00136	0.00118	0.00099	0.00119	0.00103	0.00116	0.00164	0.00249	0.00272	0.00301	0.00180	0.00245
TAG56:5(22:1)	0.00075	0.00086	0.00031	0.00052	0.00029	0.00075	0.00094	0.00051	0.00091	0.00105	0.00048	0.00094
TAG56:5(20:0)	0.00054	0.00055	0.00031	0.00060	0.00015	0.00033	0.00039	0.00081	0.00015	0.00049	0.00028	0.00043
TAG56:5(20:1)	0.00109	0.00118	0.00115	0.00097	0.00125	0.00100	0.00226	0.00293	0.00204	0.00161	0.00228	0.00209

TAG56:5(20:2)	0.00068	0.00086	0.00054	0.00149	0.00118	0.00066	0.00226	0.00183	0.00204	0.00259	0.00180	0.00252
TAG56:5(20:3)	0.00109	0.00118	0.00115	0.00149	0.00155	0.00091	0.00320	0.00381	0.00272	0.00231	0.00325	0.00317
TAG56:5(18:1)	0.00252	0.00228	0.00344	0.00246	0.00287	0.00290	0.00701	0.00688	0.00620	0.00664	0.00801	0.00655
TAG56:5(18:2)	0.00163	0.00157	0.00191	0.00112	0.00243	0.00232	0.00452	0.00329	0.00416	0.00398	0.00421	0.00432
TAG56:5(18:3)	0.00068	0.00126	0.00130	0.00082	0.00066	0.00116	0.00156	0.00066	0.00121	0.00098	0.00152	0.00086
TAG56:4(20:1)	0.00156	0.00220	0.00283	0.00231	0.00133	0.00166	0.00436	0.00388	0.00340	0.00489	0.00380	0.00288
TAG56:4(20:2)	0.00136	0.00094	0.00153	0.00119	0.00125	0.00091	0.00397	0.00417	0.00197	0.00328	0.00325	0.00216
TAG56:4(18:1)	0.00347	0.00503	0.00375	0.00463	0.00354	0.00423	0.00810	0.00820	0.00824	0.01181	0.00863	0.00742
TAG56:4(18:2)	0.00122	0.00189	0.00306	0.00269	0.00199	0.00207	0.00577	0.00505	0.00620	0.00650	0.00608	0.00569
TAG56:3(22:0)	0.00082	0.00118	0.00176	0.00037	0.00118	0.00017	0.00094	0.00103	0.00106	0.00133	0.00111	0.00187
TAG56:3(22:1)	0.00150	0.00165	0.00061	0.00127	0.00074	0.00166	0.00148	0.00168	0.00098	0.00182	0.00124	0.00173
TAG56:3(20:0)	0.00088	0.00228	0.00107	0.00082	0.00140	0.00216	0.00444	0.00381	0.00348	0.00447	0.00456	0.00310
TAG56:3(20:1)	0.00258	0.00275	0.00253	0.00216	0.00346	0.00340	0.00577	0.00564	0.00688	0.00468	0.00670	0.00562
TAG56:3(18:1)	0.00700	0.00778	0.00582	0.00672	0.00553	0.00680	0.01216	0.01303	0.01361	0.01300	0.01416	0.01102
TAG56:3(18:2)	0.00136	0.00196	0.00145	0.00202	0.00169	0.00174	0.00600	0.00615	0.00431	0.00503	0.00546	0.00504
TAG56:2(22:1)	0.00041	0.00047	0.00069	0.00052	0.00037	0.00075	0.00148	0.00095	0.00098	0.00161	0.00076	0.00151
TAG56:2(20:1)	0.00109	0.00181	0.00107	0.00090	0.00147	0.00141	0.00335	0.00308	0.00363	0.00335	0.00449	0.00202
TAG56:2(18:1)	0.00721	0.00597	0.00620	0.00784	0.00619	0.01037	0.00990	0.01501	0.01187	0.01104	0.01084	0.01239
TAG58:9(22:6)	0.00122	0.00157	0.00099	0.00097	0.00155	0.00075	0.00405	0.00264	0.00454	0.00356	0.00338	0.00324
TAG58:9(22:5)	0.00054	0.00110	0.00084	0.00060	0.00059	0.00050	0.00125	0.00051	0.00061	0.00126	0.00097	0.00086
TAG58:8(22:6)	0.00136	0.00071	0.00092	0.00172	0.00169	0.00158	0.00514	0.00454	0.00431	0.00426	0.00449	0.00583
TAG58:8(22:5)	0.00353	0.00479	0.00444	0.00284	0.00508	0.00473	0.00538	0.00469	0.00492	0.00468	0.00394	0.00511
TAG58:8(20:4)	0.00150	0.00031	0.00161	0.00097	0.00125	0.00058	0.00249	0.00146	0.00151	0.00112	0.00069	0.00122
TAG58:7(20:4)	0.00136	0.00039	0.00061	0.00045	0.00015	0.00141	0.00125	0.00124	0.00136	0.00049	0.00097	0.00065

TAG58:7(22:6)	0.00150	0.00118	0.00184	0.00097	0.00162	0.00133	0.00491	0.00439	0.00469	0.00552	0.00442	0.00367
CE-14:0	0.00642	0.00796	0.00508	0.00585	0.00401	0.00427	0.02645	0.03371	0.03273	0.02708	0.03448	0.02537
CE-15:0	0.01766	0.01737	0.01547	0.01730	0.02079	0.01785	0.04115	0.04074	0.02894	0.03668	0.03668	0.03285
CE-16:2	0.00252	0.00314	0.00254	0.00468	0.00351	0.00754	0.00686	0.01309	0.00783	0.00714	0.00929	0.00773
CE-16:1	0.00711	0.00748	0.00947	0.01076	0.00902	0.00805	0.06392	0.06596	0.06712	0.06744	0.06383	0.06402
CE-16:0	0.03280	0.03040	0.02771	0.03601	0.03056	0.03394	0.44648	0.46025	0.44778	0.44183	0.46074	0.43459
CE-17:1	0.00115	0.00241	0.00185	0.00327	0.00200	0.00251	0.02082	0.01940	0.02158	0.01846	0.01785	0.01474
CE-17:0	0.00252	0.00241	0.00346	0.00257	0.00175	0.00427	0.05021	0.04995	0.05076	0.04824	0.05600	0.05363
CE-18:3	0.00413	0.00410	0.00485	0.00327	0.00326	0.00402	0.04310	0.05480	0.04293	0.04160	0.04622	0.04155
CE-18:2	0.02546	0.03668	0.03094	0.02596	0.02956	0.02137	0.69947	0.83222	0.73192	0.85634	0.83882	0.84648
CE-18:1	0.12479	0.12017	0.12609	0.12978	0.13903	0.12496	1.50842	1.61401	1.44936	1.66640	1.83293	1.53835
CE-18:0	0.01239	0.01689	0.02425	0.01567	0.01979	0.02238	0.31447	0.30748	0.32872	0.35716	0.34996	0.35149
CE-20:5	0.00275	0.00507	0.00346	0.00538	0.00476	0.00729	0.02449	0.02813	0.02609	0.02141	0.03032	0.02633
CE-20:4	0.04175	0.03981	0.03094	0.03671	0.02931	0.04148	0.24785	0.27814	0.22271	0.25279	0.25458	0.21355
CE-20:3	0.00573	0.00893	0.00531	0.00631	0.00701	0.00528	0.14597	0.14840	0.13400	0.14990	0.15700	0.17418
CE-20:2	0.00344	0.00410	0.00531	0.00491	0.00501	0.00603	0.11633	0.09093	0.09700	0.09698	0.10809	0.10364
CE-20:1	0.00413	0.00458	0.00670	0.00398	0.00451	0.00754	0.07054	0.07832	0.07803	0.08640	0.09953	0.07924
CE-20:0	0.02065	0.03402	0.02194	0.03321	0.03207	0.03671	0.05731	0.06790	0.05645	0.05612	0.05454	0.05798
CE-22:6	0.01262	0.01810	0.01270	0.01567	0.02430	0.01810	0.11805	0.12803	0.12357	0.11766	0.14111	0.13577
CE-22:5	0.01032	0.01979	0.01432	0.01894	0.01378	0.01861	0.06196	0.07153	0.05882	0.06646	0.07972	0.07223
CE-22:4	0.00849	0.01038	0.01132	0.01146	0.01353	0.01232	0.09380	0.09651	0.09392	0.10510	0.10663	0.11161
CE-22:3	0.00665	0.01062	0.00924	0.00935	0.00902	0.00880	0.02057	0.02425	0.02111	0.02117	0.02421	0.02271
CE-22:2	0.00528	0.00458	0.00346	0.00468	0.00576	0.00603	0.02817	0.02376	0.02372	0.01920	0.01834	0.02029
CE-22:1	0.00390	0.00338	0.00531	0.00234	0.00451	0.00327	0.02743	0.02861	0.03392	0.02757	0.02617	0.03044

CE-22:0	0.00390	0.00386	0.00346	0.00210	0.00376	0.00352	0.01396	0.01358	0.01044	0.01698	0.02005	0.01377
LPE16:1	0.00135	0.00169	0.00177	0.00176	0.00141	0.00174	0.00080	0.00070	0.00063	0.00104	0.00075	0.00068
LPE16:0	0.02528	0.02579	0.02562	0.02594	0.02737	0.02622	0.01673	0.01821	0.01879	0.01794	0.01734	0.01957
LPE18:2	0.00986	0.01232	0.01114	0.01178	0.01047	0.01200	0.01842	0.01861	0.01712	0.01773	0.01598	0.01873
LPE18:1	0.01726	0.01932	0.02130	0.02223	0.02259	0.02208	0.02153	0.02634	0.02342	0.02381	0.02092	0.02418
LPE18:0	0.04371	0.04996	0.04998	0.05146	0.05238	0.05393	0.03705	0.04269	0.03953	0.04181	0.03650	0.04351
PE32:2	0.00082	0.00463	0.00106	0.00168	0.00069	0.00086	0.00068	0.00115	0.00156	0.00034	0.00179	0.00060
PE32:1	0.00711	0.00772	0.00634	0.00842	0.00555	0.00402	0.00159	0.00115	0.00311	0.00169	0.00036	0.00090
PE32:0	0.01231	0.01120	0.00599	0.01449	0.01007	0.01293	0.00205	0.00143	0.00311	0.00305	0.00214	0.00151
PE34:2	0.06704	0.07105	0.07401	0.06403	0.06388	0.06034	0.04820	0.05016	0.06790	0.05484	0.04932	0.06417
PE34:1	0.03776	0.05947	0.04616	0.04819	0.04652	0.04166	0.02023	0.02407	0.03270	0.02538	0.02359	0.02500
PE34:0	0.00465	0.00849	0.00775	0.00809	0.00486	0.00402	0.00386	0.00458	0.00529	0.00135	0.00607	0.00151
PE36:4	0.27252	0.41612	0.41543	0.34954	0.34450	0.31154	0.10550	0.14881	0.13116	0.15137	0.14302	0.14887
PE36:3	0.06512	0.07761	0.07401	0.07482	0.06666	0.05717	0.04387	0.04757	0.04983	0.05077	0.03753	0.03765
PE36:2	0.09387	0.11162	0.07894	0.10686	0.11217	0.08017	0.08095	0.07998	0.10093	0.10428	0.08616	0.10938
PE36:1	0.02763	0.04131	0.02784	0.02662	0.02951	0.02700	0.02682	0.02636	0.04485	0.03182	0.03717	0.02952
PE38:7	0.00465	0.00309	0.00352	0.00337	0.00139	0.00144	0.00205	0.00143	0.00187	0.00237	0.00465	0.00241
PE38:6	0.08757	0.13286	0.13043	0.11124	0.10869	0.09915	0.03796	0.03897	0.05887	0.03926	0.04468	0.03313
PE38:5	0.25171	0.36337	0.31042	0.29888	0.30024	0.23957	0.10507	0.12616	0.14487	0.12764	0.13694	0.13591
PE38:4	0.98481	1.20741	1.18443	1.05398	1.07631	0.93121	0.38606	0.57084	0.58028	0.51148	0.58947	0.53605
PE38:3	0.09907	0.14368	0.13325	0.10112	0.10695	0.09571	0.04387	0.06707	0.06230	0.06364	0.07399	0.05874
PE40:6	0.02161	0.03205	0.02044	0.02156	0.02152	0.02528	0.01454	0.02092	0.02024	0.01895	0.02430	0.02319
PE40:5	0.01094	0.01737	0.01409	0.01280	0.01388	0.01379	0.00727	0.01146	0.00903	0.00948	0.01286	0.00903
PE40:4	0.01258	0.01853	0.01233	0.01617	0.01770	0.01235	0.00773	0.01490	0.01370	0.01015	0.01680	0.01024

PE34:2p	0.00356	0.00309	0.00846	0.00337	0.00521	0.00488	0.00205	0.00172	0.00280	0.00338	0.00429	0.00211
PE34:1p	0.00656	0.00618	0.00493	0.00606	0.00798	0.00402	0.00454	0.00401	0.00467	0.00474	0.00286	0.00422
PE36:4p	0.07964	0.08650	0.07543	0.06977	0.07222	0.07443	0.02659	0.02980	0.03706	0.03114	0.03503	0.03374
PE36:3p	0.00492	0.00733	0.00881	0.00977	0.01007	0.01178	0.00545	0.00487	0.00685	0.00474	0.00500	0.00392
PE36:2p	0.00082	0.00502	0.00458	0.00168	0.00278	0.00345	0.00205	0.00287	0.00405	0.00372	0.00429	0.00271
PE36:1p	0.00410	0.00386	0.00211	0.00303	0.00278	0.00402	0.00386	0.00401	0.00529	0.00575	0.00572	0.00632
PE38:6p	0.01778	0.01930	0.01586	0.01348	0.01527	0.01637	0.00682	0.01146	0.01681	0.01151	0.01322	0.00964
PE38:5p	0.02845	0.03784	0.03735	0.02830	0.03818	0.03447	0.01750	0.02206	0.02429	0.03216	0.02645	0.03042
PE38:4p	0.03173	0.05212	0.04828	0.04414	0.04964	0.03591	0.02727	0.05015	0.05263	0.05720	0.06184	0.04669
PE40:6p	0.00547	0.00965	0.00423	0.00741	0.00764	0.00431	0.00727	0.00917	0.00685	0.01015	0.00786	0.00903
PE40:5p	0.00492	0.00618	0.00458	0.00775	0.00659	0.00230	0.00250	0.00630	0.00623	0.00237	0.00393	0.00542
PE40:4p	0.00520	0.00347	0.00493	0.00371	0.00382	0.00345	0.00250	0.00516	0.00623	0.00440	0.00536	0.00542
PE42:3p	0.00164	0.00154	0.00070	0.00034	0.00035	0.00172	0.00068	0.00172	0.00218	0.00102	0.00322	0.00090
PE42:2p	0.00219	0.00386	0.00070	0.00270	0.00243	0.00201	0.00068	0.00029	0.00093	0.00102	0.00143	0.00030
PE42:1p	0.00137	0.00232	0.00317	0.00168	0.00208	0.00144	0.00068	0.00057	0.00062	0.00102	0.00107	0.00060
PE42:0p	0.00055	0.00193	0.00141	0.00135	0.00139	0.00057	0.00045	0.00057	0.00000	0.00068	0.00000	0.00030
LPA16:2	0.00000	0.00000	0.00001	0.00000	0.00001	0.00000	0.00000	0.00001	0.00001	0.00001	0.00000	0.00000
LPA16:1	0.00002	0.00002	0.00002	0.00002	0.00001	0.00001	0.00002	0.00001	0.00001	0.00002	0.00001	0.00002
LPA16:0	0.00027	0.00036	0.00035	0.00034	0.00035	0.00036	0.00021	0.00029	0.00024	0.00031	0.00024	0.00022
LPA18:3	0.00003	0.00005	0.00005	0.00005	0.00005	0.00003	0.00004	0.00003	0.00004	0.00003	0.00005	0.00003
LPA18:2	0.00017	0.00021	0.00021	0.00019	0.00026	0.00024	0.00020	0.00022	0.00019	0.00022	0.00024	0.00021
LPA18:1	0.00013	0.00016	0.00014	0.00017	0.00016	0.00015	0.00018	0.00019	0.00019	0.00020	0.00021	0.00021
LPA18:0	0.00023	0.00031	0.00031	0.00032	0.00038	0.00036	0.00031	0.00034	0.00031	0.00034	0.00038	0.00038
PA32:2	0.00005	0.00008	0.00009	0.00007	0.00006	0.00006	0.00005	0.00008	0.00003	0.00008	0.00009	0.00006

PA32:1	0.00015	0.00014	0.00019	0.00016	0.00015	0.00014	0.00007	0.00009	0.00006	0.00008	0.00011	0.00007
PA32:0	0.00129	0.00150	0.00161	0.00156	0.00144	0.00157	0.00058	0.00056	0.00050	0.00049	0.00062	0.00064
PA34:2	0.00098	0.00134	0.00136	0.00128	0.00131	0.00136	0.00087	0.00083	0.00085	0.00072	0.00084	0.00081
PA34:1	0.00069	0.00085	0.00091	0.00079	0.00094	0.00075	0.00071	0.00073	0.00066	0.00063	0.00077	0.00071
PA36:2	0.00079	0.00092	0.00116	0.00102	0.00106	0.00104	0.00115	0.00120	0.00115	0.00133	0.00113	0.00119
PA36:1	0.00053	0.00064	0.00075	0.00049	0.00068	0.00070	0.00084	0.00095	0.00088	0.00096	0.00085	0.00087
PA38:5	0.00055	0.00067	0.00077	0.00059	0.00076	0.00065	0.00021	0.00032	0.00024	0.00027	0.00038	0.00033
PA38:4	0.00334	0.00407	0.00469	0.00355	0.00421	0.00477	0.00217	0.00228	0.00216	0.00227	0.00184	0.00240
PA38:3	0.00054	0.00059	0.00070	0.00071	0.00073	0.00064	0.00038	0.00044	0.00043	0.00055	0.00034	0.00050
PA40:6	0.00016	0.00018	0.00021	0.00012	0.00017	0.00014	0.00018	0.00022	0.00013	0.00019	0.00021	0.00017
PA40:5	0.00005	0.00011	0.00012	0.00011	0.00009	0.00011	0.00012	0.00010	0.00009	0.00016	0.00011	0.00014
LPI16:0	0.00485	0.00604	0.00664	0.00584	0.00634	0.00582	0.00498	0.00459	0.00478	0.00443	0.00440	0.00453
LPI18:0	0.01495	0.01871	0.01742	0.01768	0.01764	0.01791	0.01934	0.01734	0.01857	0.01704	0.01668	0.01833
LPI20:4	0.02188	0.02859	0.02843	0.02843	0.02756	0.02749	0.02178	0.02233	0.02195	0.02173	0.02092	0.02190
PI 34:2	0.00491	0.00495	0.00520	0.00637	0.00678	0.00702	0.00550	0.00488	0.00753	0.00659	0.00633	0.00521
PI 34:1	0.00724	0.00746	0.00796	0.00829	0.00934	0.01001	0.00776	0.00798	0.01163	0.00946	0.00900	0.00784
PI 36:4	0.02385	0.02394	0.02587	0.02662	0.03095	0.03393	0.01666	0.01682	0.01787	0.01703	0.01773	0.01751
PI 36:3	0.00694	0.00688	0.00706	0.00744	0.00867	0.01010	0.00708	0.00774	0.00907	0.00785	0.00767	0.00704
PI 36:2	0.00903	0.00885	0.00989	0.01096	0.01178	0.01198	0.02037	0.01963	0.02460	0.01957	0.01981	0.01884
PI 36:1	0.00283	0.00305	0.00282	0.00359	0.00359	0.00400	0.00613	0.00613	0.00804	0.00762	0.00753	0.00634
PI 38:6	0.00516	0.00533	0.00492	0.00545	0.00645	0.00656	0.00348	0.00361	0.00386	0.00370	0.00307	0.00352
PI 38:5	0.01048	0.01085	0.01007	0.01099	0.01241	0.01347	0.01237	0.01276	0.01475	0.01242	0.01179	0.01416
PI 38:4	0.18758	0.19550	0.20294	0.21321	0.23663	0.24597	0.21099	0.20259	0.23097	0.21109	0.18921	0.21574
PI 38:3	0.03594	0.03877	0.04103	0.03995	0.04434	0.05160	0.04652	0.04513	0.05572	0.04701	0.04318	0.04896

PI 40:6	0.00418	0.00408	0.00453	0.00476	0.00482	0.00578	0.00376	0.00372	0.00368	0.00324	0.00327	0.00378
PI 40:5	0.00185	0.00188	0.00213	0.00237	0.00272	0.00287	0.00292	0.00283	0.00262	0.00294	0.00251	0.00285
PI 40:4	0.00232	0.00234	0.00237	0.00240	0.00269	0.00302	0.00361	0.00338	0.00342	0.00332	0.00325	0.00366
LPS16:1	0.00009	0.00011	0.00010	0.00014	0.00013	0.00008	0.00013	0.00006	0.00008	0.00011	0.00007	0.00007
LPS16:0	0.00215	0.00292	0.00309	0.00297	0.00274	0.00325	0.00219	0.00218	0.00229	0.00209	0.00228	0.00250
LPS18:1	0.00594	0.00732	0.00725	0.00769	0.00756	0.00773	0.00859	0.00924	0.00881	0.00947	0.00930	0.01040
LPS18:0	0.01696	0.02279	0.02242	0.02236	0.02309	0.02284	0.01945	0.01936	0.01817	0.01901	0.01883	0.02064
PS 34:2	0.01221	0.01446	0.01367	0.01304	0.01134	0.01281	0.01421	0.01538	0.01467	0.01399	0.01521	0.01487
PS 34:1	0.04087	0.05072	0.04658	0.04830	0.04720	0.04789	0.03130	0.03457	0.03161	0.03163	0.03375	0.03119
PS 36:2	0.11418	0.13806	0.13023	0.12627	0.12348	0.13089	0.20019	0.21225	0.19783	0.19899	0.20055	0.20768
PS 36:1	0.19594	0.23728	0.21821	0.21780	0.22044	0.22258	0.28965	0.32585	0.30602	0.30098	0.29865	0.30477
PS 38:5	0.05480	0.06801	0.06305	0.06362	0.06263	0.06269	0.04431	0.04750	0.04526	0.04299	0.04511	0.04609
PS 38:4	1.62018	2.09901	1.84354	1.91230	1.86568	1.94055	1.02183	1.08036	1.00618	0.98639	0.99365	1.03781
PS 38:3	0.23768	0.30704	0.27974	0.27283	0.27454	0.28248	0.19429	0.20537	0.18874	0.19052	0.19792	0.20184
PS 40:7	0.00298	0.00449	0.00335	0.00359	0.00322	0.00332	0.00331	0.00347	0.00347	0.00316	0.00350	0.00325
PS 40:6	0.04136	0.04919	0.04333	0.04751	0.04671	0.04751	0.05999	0.06375	0.06191	0.05643	0.05908	0.06490
PS 40:5	0.03663	0.04804	0.04386	0.04176	0.04408	0.04358	0.03992	0.04458	0.04255	0.04051	0.04134	0.04340
PS 40:4	0.06810	0.08877	0.07809	0.07963	0.07853	0.08061	0.06413	0.06991	0.06514	0.06448	0.06540	0.06668
PG32:2	0.00079	0.00030	0.00055	0.00070	0.00056	0.00114	0.00022	0.00025	0.00060	0.00073	0.00082	0.00053
PG32:1	0.00297	0.00108	0.00217	0.00235	0.00204	0.00332	0.00095	0.00114	0.00133	0.00066	0.00088	0.00145
PG34:3	0.00079	0.00030	0.00038	0.00076	0.00046	0.00082	0.00017	0.00000	0.00033	0.00060	0.00044	0.00031
PG34:2	0.00535	0.00298	0.00439	0.00413	0.00440	0.00387	0.00512	0.00538	0.00485	0.00445	0.00384	0.00443
PG34:1	0.03824	0.02625	0.02622	0.03405	0.03105	0.03124	0.02656	0.02565	0.01868	0.01549	0.02036	0.02063
PG36:4	0.01008	0.01049	0.01082	0.01183	0.01611	0.01362	0.00868	0.01108	0.01270	0.00698	0.01147	0.00856

PG36:3	0.00514	0.00505	0.00332	0.00515	0.00593	0.00556	0.00417	0.00595	0.00751	0.00472	0.00643	0.00581
PG36:2	0.00501	0.00449	0.00588	0.00750	0.00700	0.00627	0.00968	0.01108	0.01236	0.01070	0.01159	0.01100
PG36:1	0.00259	0.00255	0.00234	0.00242	0.00266	0.00212	0.00462	0.00563	0.00585	0.00326	0.00498	0.00512
PG38:6	0.00134	0.00104	0.00136	0.00159	0.00169	0.00174	0.00184	0.00127	0.00133	0.00066	0.00157	0.00176
PG38:5	0.00247	0.00112	0.00128	0.00273	0.00148	0.00109	0.00189	0.00184	0.00080	0.00106	0.00132	0.00160
PG38:4	0.00330	0.00211	0.00251	0.00413	0.00158	0.00289	0.00228	0.00348	0.00259	0.00193	0.00271	0.00252
PG38:3	0.00088	0.00039	0.00043	0.00114	0.00082	0.00082	0.00133	0.00114	0.00153	0.00146	0.00126	0.00053
LBPA32:2	0.00012	0.00046	0.00034	0.00035	0.00021	0.00077	0.00019	0.00018	0.00043	0.00084	0.00027	0.00024
LBPA32:1	0.00012	0.00027	0.00110	0.00035	0.00064	0.00098	0.00028	0.00054	0.00052	0.00098	0.00107	0.00048
LBPA34:3	0.00030	0.00037	0.00008	0.00059	0.00043	0.00011	0.00084	0.00054	0.00052	0.00042	0.00053	0.00024
LBPA34:2	0.00066	0.00091	0.00051	0.00071	0.00064	0.00044	0.00262	0.00073	0.00130	0.00252	0.00267	0.00169
LBPA34:1	0.00084	0.00027	0.00042	0.00059	0.00053	0.00098	0.00150	0.00190	0.00199	0.00210	0.00232	0.00229
LBPA36:4	0.00376	0.00502	0.00262	0.00354	0.00491	0.00459	0.01067	0.00853	0.00865	0.01049	0.00820	0.00834
LBPA36:3	0.00269	0.00356	0.00432	0.00295	0.00352	0.00601	0.01441	0.01842	0.01342	0.01623	0.01498	0.01136
LBPA36:2	0.00090	0.00155	0.00178	0.00130	0.00149	0.00273	0.01151	0.01125	0.01030	0.01343	0.01186	0.01293
LBPA36:1	0.00066	0.00027	0.00017	0.00059	0.00075	0.00098	0.00299	0.00154	0.00260	0.00266	0.00330	0.00217
LBPA38:6	0.00358	0.00265	0.00440	0.00543	0.00534	0.00689	0.00692	0.00907	0.00727	0.01343	0.00758	0.01027
LBPA38:5	0.00173	0.00310	0.00279	0.00343	0.00309	0.00481	0.00833	0.00989	0.00917	0.01287	0.01026	0.00797
LBPA38:4	0.00084	0.00100	0.00144	0.00154	0.00203	0.00077	0.00355	0.00317	0.00329	0.00574	0.00446	0.00362
LBPA38:3	0.00048	0.00064	0.00017	0.00024	0.00139	0.00033	0.00131	0.00190	0.00173	0.00224	0.00178	0.00181
FFA22:6	0.78229	1.02476	1.01825	1.09404	1.33466	1.08219	2.04725	2.13854	1.95199	1.86409	2.04307	2.06736
FFA22:5	0.17424	0.23247	0.21136	0.25003	0.24899	0.23345	0.37663	0.41499	0.34619	0.39176	0.36456	0.41594
FFA22:4	0.07445	0.11778	0.10227	0.10497	0.10952	0.10099	0.18863	0.18343	0.17290	0.19415	0.18625	0.19055
FFA20:5	0.14591	0.14986	0.15683	0.17216	0.16049	0.16968	0.20269	0.19179	0.18433	0.20382	0.19868	0.21994

FFA20:4	1.45121	1.59303	1.77063	1.74268	1.73574	1.55763	1.75444	1.73881	1.63602	1.70970	1.70430	1.68875
FFA20:3	0.14456	0.14977	0.13155	0.15786	0.15064	0.13078	0.16189	0.17343	0.15517	0.18375	0.16152	0.15931
FFA18:2	1.83331	1.82504	1.50759	1.54086	1.51435	1.74870	3.44845	3.61875	3.76712	2.89835	3.31538	3.89155
FFA18:1	0.74189	0.77286	0.71104	0.56771	0.55843	0.51434	1.09764	0.99119	1.05353	0.75148	1.04286	1.10340
FFA18:0	2.14975	2.26951	2.09508	2.35050	2.43066	2.25399	2.65675	2.54576	2.50281	2.40112	2.36948	2.60867
FFA16:1	0.41705	0.39989	0.49389	0.35646	0.41882	0.33563	0.33202	0.34259	0.40476	0.34523	0.40456	0.40621
FFA16:0	1.98143	2.03595	2.12937	2.05564	2.02928	1.73521	2.14489	2.24816	2.27284	1.92570	2.06508	2.16535
LysoPC14:0e	0.00189	0.00229	0.00221	0.00225	0.00246	0.00266	0.00247	0.00222	0.00261	0.00249	0.00218	0.00212
LysoPC16:1	0.00477	0.00615	0.00563	0.00608	0.00637	0.00637	0.00452	0.00421	0.00424	0.00395	0.00409	0.00382
LysoPC16:0	0.18044	0.21353	0.20121	0.21200	0.21320	0.22784	0.18266	0.17414	0.17342	0.19811	0.17097	0.15187
LysoPC18:3	0.00094	0.00119	0.00109	0.00117	0.00113	0.00126	0.00094	0.00100	0.00101	0.00102	0.00093	0.00088
LysoPC18:2	0.06488	0.08234	0.07278	0.08931	0.08264	0.09276	0.13132	0.12252	0.12589	0.12700	0.11074	0.11013
LysoPC18:1	0.04883	0.06036	0.05670	0.06730	0.06815	0.06371	0.08091	0.08043	0.08104	0.08200	0.07400	0.08074
LysoPC18:0	0.10393	0.11896	0.11699	0.12206	0.11529	0.12975	0.11622	0.11605	0.11536	0.12737	0.10786	0.10863
LysoPC20:5	0.00391	0.00575	0.00503	0.00634	0.00572	0.00631	0.00444	0.00393	0.00428	0.00444	0.00390	0.00385
LysoPC20:4	0.17779	0.24737	0.23003	0.27983	0.21977	0.26046	0.20310	0.20343	0.21087	0.23789	0.19817	0.18062
LysoPC20:3	0.01640	0.02264	0.02032	0.02491	0.02015	0.02385	0.02352	0.02219	0.02307	0.02447	0.02089	0.02001
LysoPC22:6	0.01233	0.01787	0.01709	0.02126	0.01614	0.01930	0.02531	0.02262	0.02421	0.02520	0.02262	0.02135
LysoPC22:5	0.00297	0.00409	0.00361	0.00505	0.00401	0.00442	0.00632	0.00560	0.00568	0.00576	0.00529	0.00532
GluCer d18:1/16:0	0.00151	0.00221	0.00193	0.00199	0.00167	0.00171	0.00602	0.00555	0.00826	0.00708	0.00603	0.00702
GluCer d18:1/18:0	0.00048	0.00037	0.00080	0.00058	0.00048	0.00049	0.00112	0.00256	0.00296	0.00194	0.00281	0.00300
GluCer d18:1/20:0	0.00054	0.00087	0.00146	0.00082	0.00051	0.00114	0.00169	0.00184	0.00338	0.00301	0.00367	0.00313
GluCer d18:1/22:0	0.00098	0.00140	0.00134	0.00136	0.00131	0.00145	0.00697	0.00841	0.00946	0.00704	0.00838	0.00862
GluCer d18:0/22:0	0.00007	0.00006	0.00006	0.00009	0.00006	0.00001	0.00021	0.00019	0.00039	0.00018	0.00043	0.00027

GluCer d18:1/24:1	0.00214	0.00296	0.00270	0.00228	0.00199	0.00329	0.01046	0.00989	0.01603	0.01009	0.01513	0.01273
GluCer d18:0/24:1	0.00379	0.00514	0.00598	0.00471	0.00432	0.00762	0.01701	0.01658	0.02433	0.02180	0.02303	0.02228
GluCer d18:1/24:0	0.00007	0.00020	0.00011	0.00017	0.00020	0.00017	0.00026	0.00040	0.00019	0.00016	0.00054	0.00037
GluCer d18:0/24:0	0.00004	0.00020	0.00013	0.00017	0.00013	0.00004	0.00055	0.00042	0.00051	0.00038	0.00014	0.00077
GalCer d18:1/22:0	0.00591	0.00794	0.00854	0.00900	0.01260	0.01053	0.00855	0.00644	0.00805	0.00694	0.00991	0.00792
GalCer d18:0/22:0	0.00018	0.00017	0.00019	0.00020	0.00039	0.00029	0.00027	0.00032	0.00014	0.00035	0.00067	0.00032
GalCer d18:1/24:1	0.11406	0.16765	0.17650	0.17063	0.28965	0.20481	0.10072	0.08321	0.08278	0.08340	0.10153	0.08796
GalCer d18:0/24:1	0.01000	0.01475	0.01540	0.01527	0.02536	0.01759	0.01309	0.00972	0.01042	0.01246	0.01140	0.00967
GalCer d18:1/24:0	0.00230	0.00370	0.00364	0.00355	0.00603	0.00362	0.00265	0.00234	0.00198	0.00184	0.00227	0.00210
GalCer d18:0/24:0	0.00023	0.00049	0.00039	0.00056	0.00074	0.00043	0.00021	0.00030	0.00041	0.00018	0.00022	0.00034
Gb3 d18:1/16:0	0.00882	0.01883	0.04257	0.02598	0.01467	0.03260	0.01402	0.01410	0.01608	0.01558	0.01229	0.01289
PC32:2	0.01779	0.01273	0.01978	0.01427	0.01415	0.01005	0.01050	0.01325	0.01119	0.01150	0.01585	0.01037
PC32:1	0.12196	0.05325	0.05614	0.14108	0.15567	0.11513	0.05332	0.07905	0.07830	0.06560	0.08066	0.05494
PC32:0	0.80866	0.86321	0.67350	0.93934	1.24945	0.95503	0.26724	0.71477	0.65272	0.63936	0.65376	0.65009
PC34:3	0.05421	0.03338	0.04581	0.05282	0.04820	0.04321	0.03954	0.06319	0.03081	0.03538	0.02701	0.03290
PC34:2	0.72075	0.75660	0.79130	0.49772	1.03963	0.62100	0.84502	1.71402	0.80716	0.31164	1.02160	0.90147
PC34:1	0.67247	0.63825	0.73298	0.76069	1.08024	0.72894	0.70690	1.35782	1.05424	0.64953	0.95049	1.04459
PC36:5	0.09198	0.07859	0.14544	0.14584	0.20038	0.14140	0.06431	0.09822	0.08282	0.05735	0.08768	0.06289
PC36:4	2.67290	2.11671	3.20632	3.39312	3.95436	1.79191	1.67020	2.51637	1.28621	0.97931	2.13738	1.62450
PC36:3	0.86980	0.60041	0.73328	0.88111	0.83516	0.91268	0.64078	1.21510	0.92237	0.88304	0.91538	0.70390
PC36:2	0.89081	0.93790	0.56631	0.94295	0.91781	0.84493	1.42068	1.69060	1.70166	0.87391	1.46831	1.35108
PC36:1	0.24104	0.19214	0.23793	0.31628	0.61037	0.21489	0.47263	0.62980	0.44097	0.36634	0.47749	0.37213
PC38:6	0.83085	0.67141	0.88438	1.04597	1.07676	0.70081	0.77612	0.99121	0.84975	0.55253	0.35002	0.60827
PC38:5	0.69737	0.51444	0.36663	0.77382	0.94467	0.31997	0.61601	0.79997	0.45039	0.33641	0.54249	0.48526

PC38:4	1.96922	2.10522	1.54824	2.10163	1.07594	1.01058	1.26861	1.50763	1.21653	0.83366	1.53673	1.11055
PC38:3	0.31201	0.26448	0.25887	0.32646	0.31995	0.24145	0.31071	0.39430	0.40878	0.23985	0.31653	0.26646
PC40:7	0.03489	0.04131	0.06559	0.05824	0.07712	0.05369	0.05856	0.08213	0.05653	0.05071	0.06716	0.05203
PC40:6	0.13517	0.05046	0.15023	0.15519	0.19812	0.14728	0.18324	0.27573	0.13051	0.13548	0.16438	0.14782
PC40:5	0.06030	0.04242	0.06399	0.06726	0.07486	0.06302	0.07251	0.12142	0.06064	0.06531	0.08283	0.05365
PC32:2e	0.00695	0.00368	0.00437	0.00591	0.00677	0.00502	0.00755	0.01184	0.00667	0.00826	0.00360	0.00357
PC32:1e	0.05218	0.06308	0.06210	0.05841	0.06727	0.05513	0.03790	0.08615	0.07301	0.05455	0.08894	0.05478
PC34:2p	0.00135	0.00123	0.00131	0.00164	0.00103	0.00057	0.00099	0.00284	0.00177	0.00177	0.00072	0.00308
PC34:1p	0.01456	0.00882	0.01294	0.01591	0.02113	0.01249	0.03232	0.03787	0.02532	0.02211	0.02611	0.01070
PC34:0p	0.03083	0.02746	0.02313	0.02526	0.03056	0.02369	0.07891	0.09017	0.09420	0.06899	0.07328	0.05430
PC36:4p	0.00745	0.00357	0.00218	0.00344	0.00574	0.00445	0.00427	0.00521	0.00628	0.00472	0.00504	0.00664
PC36:3p	0.03066	0.02847	0.03069	0.04036	0.04369	0.03086	0.04905	0.05917	0.05416	0.03199	0.05113	0.04425
PC36:2p	0.00999	0.00704	0.00815	0.00903	0.01107	0.01034	0.01969	0.01822	0.01766	0.02079	0.02143	0.01507
PC36:1p	0.01711	0.01284	0.01600	0.01887	0.04041	0.01952	0.04134	0.06864	0.04592	0.04658	0.05707	0.04376
PC36:0p	0.01321	0.00637	0.01120	0.01345	0.01641	0.01306	0.03035	0.04402	0.02845	0.02727	0.03439	0.02577
PC38:4p	0.04353	0.02568	0.03097	0.05217	0.04882	0.03546	0.08121	0.10579	0.05691	0.09199	0.09219	0.10211
PC38:3p	0.04709	0.04779	0.04508	0.06414	0.06727	0.07551	0.06775	0.08757	0.07418	0.06708	0.09290	0.09547
PC38:2p	0.00897	0.01172	0.01599	0.01476	0.01539	0.01077	0.02182	0.02604	0.02178	0.01798	0.02467	0.01961
PC40:4p	0.00881	0.00614	0.00756	0.01083	0.01313	0.00933	0.01378	0.02438	0.01413	0.01415	0.01944	0.01702
PC40:3p	0.01169	0.01273	0.01134	0.01181	0.01457	0.01048	0.01198	0.02604	0.01492	0.00885	0.01837	0.01264
PC40:2p	0.00271	0.00369	0.00392	0.00328	0.00944	0.00531	0.00230	0.00852	0.00608	0.00811	0.00721	0.00551
PC40:1p	0.00762	0.00614	0.00800	0.01017	0.00861	0.00660	0.00164	0.00520	0.00334	0.00177	0.00306	0.00453
GM3 d18:1/16:0	0.00160	0.00184	0.00195	0.00207	0.00179	0.00212	0.00352	0.00489	0.00443	0.00440	0.00433	0.00502
GM3 d18:0/16:0	0.00031	0.00045	0.00027	0.00044	0.00055	0.00052	0.00094	0.00105	0.00113	0.00109	0.00135	0.00154

GM3 d18:1/18:1	0.00002	0.00004	0.00003	0.00001	0.00004	0.00004	0.00008	0.00023	0.00008	0.00023	0.00004	0.00020
GM3 d18:1/18:0	0.00083	0.00075	0.00091	0.00076	0.00065	0.00097	0.00170	0.00202	0.00195	0.00160	0.00203	0.00234
GM3 d18:0/18:0	0.00064	0.00044	0.00050	0.00055	0.00054	0.00064	0.00084	0.00111	0.00082	0.00096	0.00081	0.00104
GM3 d18:1/20:1	0.00003	0.00006	0.00008	0.00009	0.00015	0.00010	0.00014	0.00020	0.00016	0.00010	0.00015	0.00020
GM3 d18:1/20:0	0.00083	0.00109	0.00091	0.00121	0.00090	0.00102	0.00180	0.00195	0.00231	0.00190	0.00193	0.00256
GM3 d18:0/20:0	0.00026	0.00033	0.00030	0.00028	0.00024	0.00027	0.00053	0.00070	0.00080	0.00057	0.00052	0.00054
GM3 d18:1/22:1	0.00023	0.00031	0.00019	0.00042	0.00036	0.00037	0.00054	0.00085	0.00051	0.00079	0.00054	0.00064
GM3 d18:1/22:0	0.00166	0.00246	0.00247	0.00242	0.00249	0.00249	0.00510	0.00548	0.00547	0.00506	0.00565	0.00582
GM3 d18:0/22:0	0.00045	0.00050	0.00055	0.00057	0.00060	0.00043	0.00094	0.00144	0.00112	0.00143	0.00122	0.00152
GM3 d18:1/24:1	0.00211	0.00217	0.00195	0.00250	0.00219	0.00220	0.00501	0.00515	0.00564	0.00518	0.00485	0.00585
GM3 d18:1/24:0	0.00529	0.00543	0.00552	0.00588	0.00518	0.00608	0.00939	0.01160	0.01070	0.01046	0.00996	0.01223
GM3 d18:0/24:0	0.00103	0.00127	0.00120	0.00141	0.00103	0.00121	0.00226	0.00259	0.00222	0.00214	0.00199	0.00267
TAG	0.74041	0.86234	0.92850	0.88459	0.85823	0.92562	2.04578	1.96868	2.03153	1.89711	1.93182	1.96948
DAG	1.93708	2.37895	2.13338	2.00351	2.12810	2.22923	2.44802	2.52727	2.75896	2.68521	2.56150	2.53478
CE	0.23076	0.20924	0.21494	0.20708	0.29193	0.21202	4.73970	5.08691	4.77779	5.23206	5.14568	5.82499
Cho	9.59212	11.30316	10.11699	11.03318	10.89129	10.93784	10.86893	12.38159	11.65014	11.97486	11.62947	12.06884
FFA	9.89609	10.57093	10.32785	10.39291	10.69159	9.86257	14.41126	14.58745	14.44766	12.86914	13.85573	14.91702
PC	11.20217	9.97291	10.54672	12.61381	13.87284	8.91596	9.47687	14.26460	10.24156	7.08691	10.89575	9.53291
PCe/p	0.31471	0.27644	0.29493	0.35945	0.42129	0.32860	0.50282	0.70767	0.54479	0.49696	0.61956	0.51882
PE	2.26068	2.97779	2.77196	2.52641	2.55950	2.22334	1.04919	1.39174	1.50911	1.38284	1.47239	1.38956
PG	0.07894	0.05814	0.06167	0.07851	0.07578	0.07450	0.06749	0.07389	0.07046	0.05271	0.06767	0.06426
PA	0.00910	0.01109	0.01256	0.01045	0.01161	0.01192	0.00735	0.00781	0.00718	0.00773	0.00729	0.00787
PS	2.42494	3.10508	2.76363	2.82664	2.77785	2.87491	1.96315	2.10299	1.96336	1.93006	1.95417	2.02248
PI	0.30230	0.31387	0.32678	0.34239	0.38118	0.40632	0.34714	0.33720	0.39377	0.35183	0.32434	0.35545

LBPA	0.01665	0.02009	0.02014	0.02161	0.02497	0.03040	0.06512	0.06766	0.06119	0.08394	0.06929	0.06342
LPC	0.61907	0.78254	0.73267	0.83756	0.75503	0.83868	0.78172	0.75833	0.77168	0.83970	0.72163	0.68935
LPE	0.09745	0.10908	0.10981	0.11317	0.11423	0.11598	0.09453	0.10655	0.09950	0.10233	0.09148	0.10667
LPS	0.02515	0.03314	0.03285	0.03317	0.03352	0.03389	0.03036	0.03083	0.02936	0.03068	0.03048	0.03361
LPA	0.00086	0.00110	0.00109	0.00109	0.00122	0.00116	0.00095	0.00109	0.00098	0.00113	0.00113	0.00107
LPI	0.04168	0.05334	0.05248	0.05195	0.05154	0.05122	0.04610	0.04426	0.04530	0.04321	0.04200	0.04477
SM	5.52056	6.05647	4.43333	6.75744	3.88025	6.60621	4.11895	4.68685	6.53676	17.64339	5.26112	4.63447
Cer	0.09093	0.03949	0.11957	0.03860	0.06535	0.04823	0.20771	0.09395	0.28896	0.33159	0.07342	0.11053
GluCer	0.14230	0.20811	0.21916	0.21137	0.34544	0.25319	0.16976	0.14817	0.16929	0.15684	0.18616	0.16650
Gb3	0.00882	0.01883	0.04257	0.02598	0.01467	0.03260	0.01402	0.01410	0.01608	0.01558	0.01229	0.01289
GM3	0.01529	0.01714	0.01684	0.01861	0.01671	0.01847	0.03279	0.03927	0.03733	0.03590	0.03538	0.04218
